# A Molecular Dynamics Study of Vasoactive Intestinal Peptide Receptor 1 and the Basis of Its Therapeutic Antagonism

**DOI:** 10.3390/ijms20184348

**Published:** 2019-09-05

**Authors:** Dorota Latek, Ingrid Langer, Krystiana A. Krzysko, Lukasz Charzynski

**Affiliations:** 1 Faculty of Chemistry, University of Warsaw, 02-093 Warsaw, Poland; 2 Institut de Recherche Interdisciplinaire en Biologie Humaine et Moléculaire (IRIBHM), Université libre de Bruxelles, B-1070 Brussels, Belgium; 3 Faculty of Physics, University of Warsaw, 02-093 Warsaw, Poland

**Keywords:** G protein-coupled receptors, vasoactive intestinal peptide receptor 1, VPAC1, VIPR1, VIP, PACAP, homology modeling, molecular dynamics, GPCRM, gut hormone receptors, GPCR activation, antagonist, agonist

## Abstract

Vasoactive intestinal peptide receptor 1 (VPAC1) is a member of a secretin-like subfamily of G protein-coupled receptors. Its endogenous neuropeptide (VIP), secreted by neurons and immune cells, modulates various physiological functions such as exocrine and endocrine secretions, immune response, smooth muscles relaxation, vasodilation, and fetal development. As a drug target, VPAC1 has been selected for therapy of inflammatory diseases but drug discovery is still hampered by lack of its crystal structure. In this study we presented the homology model of this receptor constructed with the well-known web service GPCRM. The VPAC1 model is composed of extracellular and transmembrane domains that form a complex with an endogenous hormone VIP. Using the homology model of VPAC1 the mechanism of action of potential drug candidates for VPAC1 was described. Only two series of small-molecule antagonists of confirmed biological activity for VPAC1 have been described thus far. Molecular docking and a series of molecular dynamics simulations were performed to elucidate their binding to VPAC1 and resulting antagonist effect. The presented work provides the basis for the possible binding mode of VPAC1 antagonists and determinants of their molecular recognition in the context of other class B GPCRs. Until the crystal structure of VPAC1 will be released, the presented homology model of VPAC1 can serve as a scaffold for drug discovery studies and is available from the author upon request.

## 1. Introduction

**Figure 1:**
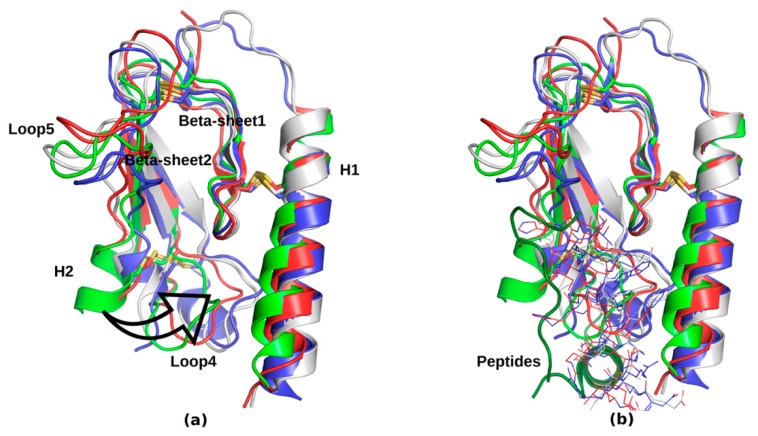
(**a**) Comparison of extracellular domains in experimental structures of glucagon receptor (GCGR, blue, PDB id: 5IQZ), glucagon-like peptide-1 receptor (GLP-1R, grey, PDB id: 5VAI), parathyroid hormone receptor PTHR1 (red, PDB id: 6NBF) and calcitonin gene-related peptide receptor (CGRP, green, PDB id: 6E3Y). The least diverse are regions of a helix H1 and a β-sheet 1. The most structural diversity (a black arrow) between these ECD domains of class B GPCRs can be observed in the region of a helix H2 and a loop 4, which is involved in the binding of peptide agonists (**b**). Similar helical conformations of peptide analogs of GCG, GLP and PTH were shown with the ‘lines’ representation while a different conformation of CGRP agonist (dark green) was shown with the ‘cartoons’ representation. The helical part of the CGRP agonist is located perpendicularly to other class B agonists because of additional interactions with receptor activity-modifying protein (RAMP).

Class B G protein-coupled receptors (GPCRs) constitute the second (after class A), studied in detail, branch of a GPCR phylogenetic tree [1]. A peptide-driven activation of class B GPCRs leads to either increase in cyclic adenosine monophosphate (cAMP) effector molecules concentrations or translocation of arrestin to the plasma membrane. With high concentrations of class B agonists also Gq-induced intracellular calcium response is possible [2]. Interestingly, class B endogenous peptides have proved to have little selectivity for various GPCR receptors, e.g., secretin binds not only to SCTR but also to vasoactive intestinal peptide receptor 1 (VPAC1) and VPAC2 [3], but with much lower potency (1000-fold and 10000-fold, respectively). Other primary examples of such low selectivity in the secretin-like GPCR subfamily were observed for growth hormone–releasing hormone (GHRH) [4] and corticotropin-releasing factor (CRF) [5,6]. The mostly studied class B GPCRs thus far are: secretin receptor SCTR, pituitary adenylate cyclase-activating polypeptide type 1 receptor (PAC1R), calcitonin receptor (CTR), corticotropin-releasing factor receptors CRFR1 and CRFR2, glucose-dependent insulinotropic polypeptide receptor also known as gastric inhibitory polypeptide receptor (GIPR), glucagon receptor (GCGR), glucagon-like peptide-1 receptor (GLP1R), growth-hormone-releasing hormone receptor (GHRHR), parathyroid hormone receptors 1 and 2 (PTHR1 and PTHR2), vasoactive intestinal peptide receptors 1 and 2 (VPAC1 and VPAC2). Other class B GPCRs include: amylin receptors (AMY_1–3_), calcitonin gene-related peptide receptor (CGRP) and adrenomedullin receptors (AM_1–2_). Among class B receptors, five were characterized structurally by experimental methods (X-ray/cryo-EM): GCGR (as a first one, in 2013) [7], GLP1R in 2017 [8], CRFR1 in 2013 [9], CTR in 2017 [10], and CGRP and PTHR1 in 2018 [11,12]. These findings provided basis for understanding the class B negative allosteric modulation (GCGR, GLP1R, CRFR1) and relative conformational changes between two subdomains of these GPCRs induced by peptide agonists during activation (GCGR, GLP1R, CTR, PTH1R, CGRP) [1]. Except for similar to the class A mode of activation involving among others the transmembrane helix 6 (TMH6) transformation, secretin-like receptors structures undergo additional rearrangements of their extracellular domains (ectodomains) leading to the opening of the receptor interior (see Figure S1). The scale of such conformational rearrangements of an ectodomain (an extracellular domain, ECD) versus a transmembrane domain (TMD) is receptor-specific (see Figure S2). For example, ECD of calcitonin receptor-like receptor (CLR) that interacts with receptor activity-modifying protein (RAMP) forming the CGRP receptor is almost perpendicular to the TMD axis during the activation. On the contrary, ECD of PTHR1, which requires only parathyroid hormone (PTH) for activation, is parallel to the TMD axis (see Figure S2). Additionally, the conformation of the peptide agonist of CGRP differs from conformations of other peptide agonists observed in the active structures of PTHR1, GLP-1R, and GCGR (partly unfolded versus helical, respectively, see Figure 1). Nevertheless, conformations of TMD domains in solved-to-date experimental structures of class B GPCRs in their fully activated states together with G protein subunits are similar (see Figure S2). More structural differences are observed in the relative positions of ECD and TMD domains, as was mentioned above, and in the ECD domains themselves (see Figure 1 and Figure S3). Although the helix H1 and the subsequent β-sheet 1, both joined with a disulphide bridge, are very similar in class B receptors, there are some differences in other regions (see Figure 1a). The region of H2 and the loop 4, which is involved in the peptide binding in all presented structures, is varied the most. These structural differences could be associated with the peptide binding specificity in class B. The location of the ECD binding site remains the same in all known-to-date structures of class B GPCRs (see Figure 1b).

Vasoactive intestinal peptide (VIP), a highly conserved in evolution [13] endogenous peptide of VPAC1 and VPAC2 receptors, was at first classified as a gut hormone, but later studies have shown it is also distributed in peripheral and central nervous systems as a neurotransmitter [14]. VIP and its receptor VPAC1 play important roles in neuro-immuno-gastroenterology due to its effect on smooth muscles relaxation, intestinal motility, vasodilation, electrolyte secretion, and innate and adaptive immunity [15]. VPAC1 is also used in imaging cancer tumors [16]. Due to the wide spectrum of biological activity in the gut-brain axis VIP and VPAC1 have been selected for pharmacotherapy of neurodegenerative disorder, schizophrenia, asthma, diabetes, gastrointestinal motility disorder, and to treat chronic inflammatory diseases [17] such as rheumatoid arthritis and Crohn’s disease [14]. Anti-inflammatory activity of VIP is mostly due to activation of protein kinase A (PKA) that leads to reducing of pro-inflammatory cytokines [18]. 

Apart from VIP, VPAC1 and VPAC2 receptors bind also with similar affinity another peptide hormone—pituitary adenylate cyclase-activating peptide (PACAP). PACAP is present in two isoforms PACAP-27 and PACAP-38 (27 and 38-residues long, respectively), the latter one with the slightly higher affinity for PAC1 receptor. PACAP, as a master regulator of the stress response [19], has focused attention in various neurological disorders, e.g., Parkinson’s disease, migraine and post-traumatic stress disorder. It also affects cell proliferation, secretion, motility, gut immunology, blood flow, and inflammatory reactions [20]. Recent studies emphasize its role in maintaining of the energy homeostasis via autonomic nervous system (cold stress) [21] and in premature aging caused by its deficiency [22]. 

**Figure 2:**
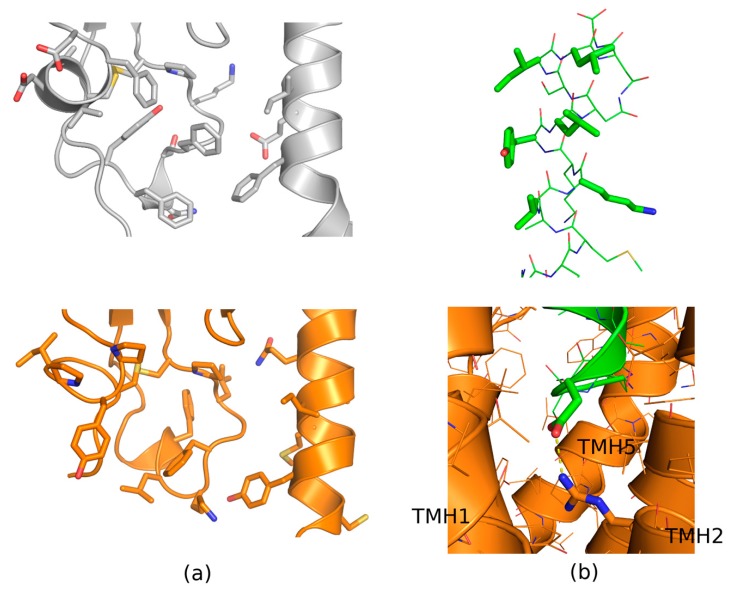
Details of interactions between vasoactive intestinal peptide receptors (VPAC receptors) and vasoactive intestinal peptide (VIP). (**a**) Comparison of ectodomain binding sites for VIP in VPAC1 (orange) and VPAC2 (grey). Most of residues involved in the peptide binding are non-polar or aromatic. (**b**) The upper part: C-terminal VIP residues which are involved in the interactions with ECD. Most of them are hydrophobic residues. The bottom part: the transmembrane domain binding site of N-terminal VIP residues. A hydrogen bond between Asp3 of the peptide and Arg188 of the receptor was observed (a yellow dashed line), similarly to the reported site-directed mutagenesis data [24]. Figures refer to the VPAC1 model (including VIP) based on the 5VAI template structure.

VPAC1, like other class B GPCRs, is composed of two subdomains: a seven transmembrane helices bundle and an extracellular part forming the N-terminal cup of the receptor closing upon deactivation (see Figure S1 for the full VPAC1 model and Figure S3 for ECD). The use of photolabeling probes combined with site-directed mutagenesis studies for the ECD domain has revealed that there are five residues (Asp107, Gly116, Cys122 and Lys127 and Gln135, residue numbering according to the Uniprot entry P32241) in ECD important for the peptide binding and the receptor activation and three other residues which are the site for the glycosylation (Asn58, Asn69, Asn100) [14]. These residues were depicted in our model of VPAC1 constructed using the recently released class B receptors structures (see Figure S4 and Figure 1). Before releasing these class B receptors structures describing the detailed interaction site for peptide ligands attempts have been made to construct the VIP-VPAC1 interactions model [23]. This model, however, included the C-terminus of VIP close to Lys127 and N-terminus close to Asp107. In contrast, new class B structures suggest that the middle of VIP should be rather close to Lys127 and its N-terminus should be inside the TMD domain interacting with Arg188, as suggested by Solano et al. [24]. Our model of VPAC1 confirms the latter hypothesis of VPAC1-VIP interactions (see Figure 2).

VPAC1 lacks a common class A sequence motif E/DRY which is substituted by a corresponding sequence motif YL that does not rather play similar role in a class B activation such as E/DRY in a class A activation. Experimentally confirmed sequence motifs of VPAC1 important for its activation are proline residues in TMH4 (P266), TMH5 (P300), and TMH6 (P348) [25] and an interaction network between TMH2 (R188), TMH3 (N229), and TMH7 (Q380), with the latter residue acting as a switching residue upon activation [26]. Site-directed mutagenesis studies have also shown that His178 (TMH2) mutated to arginine leads to a constitutively activated VPAC1 receptor. Other residues that were shown to stabilize an active conformation of VPAC1 are: Y146 and Y150 (TMH1), K195 (TMH2), and D196 (EC1); K143, T144, and T147 (TMH1). The latter three residues were proved to be involved in the peptide binding [27]. In the case of VPAC2, fewer residues were confirmed as important for activation: Y130 and Y134 (TMH1); K179 (TMH2); N216 (TMH3), but it was suggested that VPAC1 and VPAC2 share a similar pattern of activation upon the peptide binding [14]. Depending on the signaling pathway that is launched, VPAC1 interacts with G protein (or arrestin) through various residues networks located in the intracellular part of the transmembrane bundle. In the case of cAMP, residues K322 (ICL3) and E394 (TMH7) interact with the G alpha subunit in a direct (K322) or indirect way. For the intracellular calcium response, there are two fragments of ICL3, highly conserved among other class B GPCRs: I328-R329-K330-S331 (proximal) and R338-L339 (distal), crucial for the interactions between VPAC1 and G alpha. 

In this manuscript, we performed a series of molecular dynamics simulations to provide basis for the VPAC1 antagonism. Namely, we studied VPAC1 interactions with experimentally confirmed antagonists. Very few non-peptide antagonists identified in 2012 by Harikrishnan et al. [28] constitute two series of compounds: biaryl and thiophene derivatives. To our knowledge, there is no data describing their exact VPAC1 binding site. In this manuscript, we evaluated with molecular dynamics simulations possible binding sites of these compounds series to VPAC1. 

## 2. Results

### 2.1. Little Ligand Selectivity of VPAC1 and Other Class B GPCRs

A distinct feature of class B GPCRs is the possibility of their activation by various peptide hormones. For example, VIP can activate not only VPAC1 and VPAC2 but also some splice variants of PAC1 receptor (PAC1R) [29] (see Table 1). PACAP activates PAC1R and also VPAC receptors. Secretin can bind VPAC1 and VPAC2 but with much lower affinity than SCTR. The described above findings explain difficulties in the pharmacology of class B GPCRs, e.g., lack of highly selective antagonist against VPAC2 and PAC1 receptors for many years [30,31]. 

A few of class B GPCRs requires receptor-activity modifying proteins (RAMPs) for cell surface targeting and/or ligand selectivity, e.g., CTR and CLR receptors [32]. In such cases, a transmembrane helix of RAMP interacts with the transmembrane core of GPCR leading to the fully functional CGRP receptor 1 (CLR+RAMP1) and adrenomedullin receptors (AM_1_ – CLR+RAMP2 and AM_2_ – CLR+RAMP3). RAMPs can also change the ligand binding of CTR and amylin receptors (AMY_1-3_ corresponding to CTR associated with RAMP1-3) [33,34]. In the case of VPAC1, its interaction with RAMP does not alter its ligand binding but modifies its signaling pathway by enhancement of phosphoinositide hydrolysis [35]. In another study, it was shown that the interaction between VPAC2 and RAMP1 or RAMP2 leads to enhanced coupling to Gα_i/o_ [36].

The lack of specificity in ligand binding observed for the PACAP receptor subfamily can be explained by the proposed model of the evolution of their peptides (see Figure S5). It was suggested that PTH/PACAP/glucagon-like ancestral peptides evolved from one common ancestor [37], but its evolution pathway is still missing some details. The PACAP sequence has nearly not been changed [38] in comparison with its most ancient (to date) forms identified in cephalochordate and urochordate which suggest that its biological function is essential [37]. A similar common evolutionary model involving a common ancestral receptor and two rounds of genome duplication (2R) followed by teleost-specific genome duplication (3R) in some species has been proposed for the PACAP receptor subfamily with VPAC1, VPAC2, PAC1 diverged at the beginning and SCTR, GHRHR, PRPR (a receptor for PACAP-related peptide) much later [13,37].

There was a significant discrepancy between experimental data regarding the VIP/PACAP binding site in the extracellular domains of PACAP and VPAC receptors. Released in 2007, an NMR structure of the ECD domain of PAC1R with PACAP-38 (PDB id: 2JOD) [47], differs completely, in terms of topology and the location of the peptide binding site, from released in 2010 the X-ray structure of the ECD domain of PAC1R [48]. The latter experimental structure seems to be more probable due to its agreement with the mutagenesis data and the sequence and structure similarity shared among all other members of class B GPCRs [48]. Namely, this X-ray structure is similar to all experimentally solved ECD structures of other members of class B GPCRs (CRFR1, CRFR2, GLP1R, GIP1R, PTH1R and VPAC2). It also perfectly supports the common two-domain binding model of ligand/receptor interaction that has further been confirmed in all experimental structures available thus far of full-length receptors bound to their peptide ligands (GLP-1R, CGRPR, PTH1R). In this model, the ECD domain of the receptor is the principal binding site for the central and the C-terminal regions of the natural ligand (organized as an alpha helix) and ensures correct ligand positioning as well as binding selectivity, whereas binding of residues 1–6 of the ligand to the extracellular loops and TM helices drives the receptor activation. Interestingly, in all structures available, except for the mentioned above NMR structure of the ECD domain of PAC1R, the alpha helix of the peptidic ligands always fits into the binding pocket along with the N-terminal alpha helix of ECD. Finally, the overlay of the X-ray structure of the ECD domain of PAC1R with the VPAC2 ECD structure is very similar. These two receptors have the highest sequence identity with VPAC1 and also share the same natural ligands VIP and PACAP. For all these reasons, the PAC1R crystal structure from 2010 and not the PAC1R NMR structure from 2007, was selected (together with the experimental VPAC2 structure) as the basis of the current study of VPAC1. 

### 2.2. Insights Into the Mechaninism of Action of VPAC1 Antagonists

#### 2.2.1. Experimental Data for the Biological Activity of VPAC1 Antagonists

**Figure 3:**
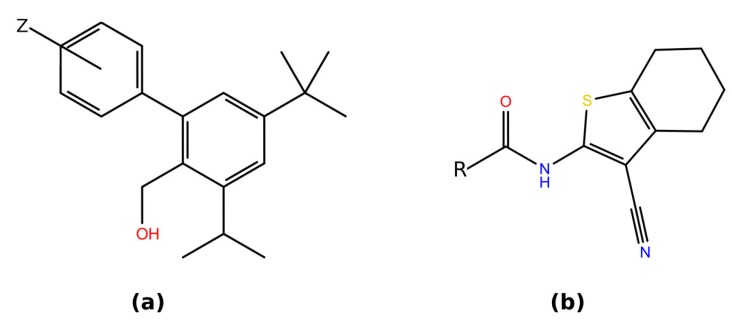
Two series of small-molecule antagonists of VPAC1 described in Harikrishnan et al.: (**a**) compound **31**, Z = 3,4-dimethyl; 3-Cl; naphthyl (3,4-fused); 3-SMe; naphthyl (2,3-fused); 3-OMe; 3-Ph; 3-Me; 4-Cl; 4-Me; 3-NMe_2_; H; 4-Ph; 4-F; 2-OMe; 4-OMe; 2-Ph; 3-CN; 3-CF_2_; 2-Me (**b**) compound **41**, R = phenylethyl; cyclohexyl; cyclopentyl; (4-methoxyphenyl)ethyl; (4-methylphenyl)ethyl; (3-methoxyphenyl)ethyl; (3-methylophenyl)ethyl; (4-chlorophenyl)ethyl; phenylmethyl; cyclobutyl; isobutyl; (2-benzo[b]thiophene)methyl; cyclopropyl; isopropyl; 2-(2,3-dihydrobenzofuran)ethyl; phenoxymethyl; (2-methylphenyl)ethyl; Et; 3-tetralin; Me; Phe; 2-indane; 2-indolemethyl. Here, tested derivatives were sorted in the ascending cAMP IC50 order (the first one—the highest potency for VPAC1).

There is little literature data on non-peptidic VPAC1 antagonists except for Harikrishnan et al. published in 2012 [28]. A total number of 53 compounds were tested in this study for their potency in activating the cAMP signaling pathway of VPAC1 and in inhibition of cell proliferation. They belonged to two series: biaryl (compound **31**) and cyanothiophene (compound **41**) (see Figure 3). For the biaryl compounds series, a correlation between the benzene substituent electron-donating/withdrawing properties and derivatives potency was observed by Harikrishnan et al. In the case of cyanothiophene analogs such correlation was not observed and no derivative with the increased biological activity with respect to the compound presented in Figure 3 has been found. 

In the case of compound **31**, methyl substituents increased its affinity towards VPAC1, possibly because of their electron-donating effect that could stabilize stacking interactions with aromatic amino acids with electron-withdrawing substituents [49]. Indeed, there are a few aromatic residues inside the ECD binding site (see 2.2.2) that could take part in such stacking interactions, but rather not in the allosteric TMD site.

Regarding the allosteric binding site of VPAC receptors, to our knowledge, the only experimentally confirmed NAM (a negative allosteric modulator) has been found for VPAC2 [50]. This compound is similar in shape to other NAMs of class B GPCRs (see Figure S6), but different from **31** and **41** compounds of Harikrishnan et al. These class B NAMs, e.g., PF-06372222 (GLP-1R) [8], NNC0640 and MK-0893 (GCGR) [51] (see Figure S6), share a common V-shape that blocks TMH6 and prevents its conformational changes during the activation. 

Slightly more data are available regarding peptide analogs of VIP and PACAP interacting with VPAC receptors. There are known peptide antagonists and agonists of PAC1 and VPAC1 [52,53,54] and of VPAC2 [31,55,56] that were tested experimentally and a few peptide analogs with the VPAC1/VPAC2 selectivity confirmed experimentally [57]. Nevertheless, it was clearly demonstrated that the N-terminus of the peptides were essential for receptor activation and discrimination between peptidic agonists and antagonists. Indeed, by combining photoaffinity labeling and mutagenesis studies, it was shown that the central and C-terminal part of PG97-269 (a selective VPAC1 antagonist) bind to the ECD of VPAC1 as does VIP. In contrast, the N-terminus of PG97-269 interacts with a different amino acid than does VIP (G62 for the antagonist and Q135 for VIP), and therefore, prevents the VPAC1 activation [27]. There were also attempts to use radio-labeled VIP analogs in cancer imaging [58] and only recently the basis of peptide analogs selectivity in the activation of PAC1 versus VPAC1 was described [59,60].

The major problem with endogenous VIP and PACAP peptides and their analogs is weak bioavailability, a short half-life when digested, and side effects that can be partly omitted when administered by inhalation [61,62]. To improve their transfer through bio-barriers (blood-brain BBB, blood-air BAB, blood-testis BTB), the TAT sequence (a cell-penetrating peptide CPP) was added which resulted in 2.5 fold increase in efficiency (PACAP) [37,63]. The selectivity improvement, e.g., for VPAC2 over VPAC1 (vasomera®), was reported to reduce gastrointestinal side effects associated with the excessive activation of VPAC1 [64].

#### 2.2.2. Binding Modes of VPAC1 Small-Molecule Antagonists Proposed by Molecular Docking

A single, experimentally determined structure of the VPAC2 extracellular domain (PDB id: 2X57) does not contain any small-molecule antagonist of this receptor that could be useful for drug discovery studies and there is no data on the VPAC1 structure in PDB at all. Nevertheless, ectodomains of class B GPCRs are similar structurally to each other (see Figure 1). For example, heavy-atoms RMSD of the main core of ECD of GCGR (PDB id: 5XEZ, the antagonist-bound conformational state) and the main core of VPAC2 ECD domain structure is equal to 3.18 Å. The sequence identity of these two cores of ECD domains is only 21.13% (computed with ClustalOmega [65]). ECD domains of class B GPCRs that have been solved thus far share the same α/β topology, stabilized by a number of disulphide bonds [1] (see Figure 1). For this reason, most probably, the ECD domain of VPAC1 is similar to other ectodomains of class B GPCRs and thus may include a similar binding site for small-molecule antagonists, if any. Yet, there is still very little data on such binding site except for the crystal structure of the CGRP ectodomain with small-molecule antagonists [66]. Although the CGRP activation requires interactions not only with peptide agonists, but also with RAMP; this ectodomain small-molecule binding site of this receptor is located in the same region that is occupied by peptide agonists in other, solved thus far, class B crystal structures. 

For that reason, we decided to test the corresponding ectodomain binding site in VPAC1 in a small-molecule docking experiment using VPAC1 antagonists of confirmed biological activity [28]. In addition, a potential allosteric binding site in the TMD domain of VPAC1 was also tested, because there is no data whether these antagonists bind to ECD or TMD domains of VPAC1. For that reason, a possibility that these antagonists act as allosteric negative modulators of VPAC1, though less probable, cannot be discarded based on the current knowledge. The docking of two of the most potent antagonists (see Figure 3) to the orthosteric binding site located in ECD that binds VIP (see Figure 1 and Figure 2, and Figures S7 and S9) and to the allosteric binding site located in TMD (see Figures S8 and S10) was performed. The orthosteric binding site located in the TMD domain of class B GPCRs is weakly druggable due to its fitness to more spacious peptide ligands and therefore was discarded [1]. Out of the two binding sites in ECD predicted by SiteMap [67,68] (see Table S1 and Figure S7), we selected Site1 (ranked as 1^st^), which was in agreement with known-to-date crystal structures of class B GPCRs (see above).

For a docking study we used four conformations of ECD domains of VPAC receptors: the VPAC2 ECD domain structure solved with NMR (PDB id: 2X57) and three models of the VPAC1 ECD domain that were built using the full class B receptors crystal structures (PDB id: 5NX2 – GLP-1R, 5VAI – GLP-1R, 5YQZ - GCGR) (see Figure 2 and Figure S3). In all four cases, we selected the ECD binding site in the same place where the peptide ligand is bound in the corresponding crystal structures of other class B receptors (see Figure 1). The selected ECD binding site contained a few aromatic amino acids (Tyr118, Tyr39 and Phe90) that could potentially interact with aromatic groups of VPAC1 antagonists via stacking (see Figure 2). The NMR-determined VPAC2 ECD binding site was rather flat, hardly a cavity-like, in contrast to binding sites in VPAC1 models (see Figure S9). The most spacious cavity was in the VPAC1 ECD model based on the 5VAI template and for this model also the lowest ligand binding energies were obtained in docking (see Table S2). The highest Autodock VINA [69] binding energies were obtained in the case of the flat binding site of VPAC2 (see Table S2). Results obtained for VPAC1 models based on 5NX2 and 5YQZ were comparable. In general, the ligand binding cavities in VPAC1 models based on 5NX2 and 5YQZ were composed of two minor cavities that were joined into one cavity in the case of VPAC1 model based on 5VAI (see Figure S9). These two cavities (joined in one) could fit compounds with two rings such as compound **31** and **41** (see Figure 3).

Although experimental results [28] are limited, as described in 2.2.1., they highly correspond to the obtained computational results regarding the ECD domain. In docking results involving this VPAC1 domain two major orientations of compound **41** that were fitted deeply inside the binding pocket were observed (see Table S2). The remaining orientations of compound **41** obtained with Autodock VINA represented partly docked ligands and therefore were discarded.

The pose ranked as the first by Autodock VINA (orientation 1) included the cyanothiophene (tetrahydrobenzothiophene-3-carbonitrile) ring between Tyr118 and Phe90 residues (see pose 1 in Tables S2 and S3). The other ligand orientation (orientation 2—Table S2, see pose 6, 8, 16 in Table S3) was rotated 180 degrees with the phenyl ring located between or near the mentioned above aromatic residues. In this case, the cyanothiophene ring could form polar interactions with adjacent Lys91, Tyr39 or more distant Glu36. These polar interactions of compound **41** seem to be plausible and were described for cyanothiophene derivatives (see PDB id: 2AM1 [70], 6BKW [71]) [72]. 

Experimental results of Harikrishnan et al. have shown that substituting of the ligand phenyl group with more bulky substituents, e.g., tetralin or 1H-indole, resulted in the decreased potency of compound **41** for VPAC1 [28]. These results suggest that the phenyl ring of compound **41** should rather be located deep in the pocket (orientation 2) rather than on the surface. 

The obtained docking results for compound **41** provided insights into its binding mode. Based on them, it could be concluded that to improve the compound **41** potency for VPAC1, stacking interactions [49] between its phenyl ring and Tyr118/Phe90 should be enhanced but without any significant increasing of this group size. In [28] a number of substituents have been already tested: methyl groups (orto, para, meta), methoxy groups (meta, para), and chloride (para), but still no compound of better potency than **41** have been found. The largest decrease of the compound **41** potency was observed in the case of the orto-methylphenyl group and orto-chlorophenyl and the least decrease in the case of para-methoxyphenyl and para-methylphenyl groups. Most probably, substituents located in orto or meta positions cause steric clashes, while para positions are more favorable sterically. Substituents that are electron-donating (CH3, OCH3) caused the least decrease of the compound **41** potency comparing the electron-withdrawing substituent (Cl). Another conclusion that could be driven based on the study of Harikrishnan et al. [28] is that a short, flexible fragment ending with the phenyl ring should be present in VPAC1 antagonists, because shortening of this fragment or replacing it with a larger rigid ring decreased the ligand potency. The docking results described in this manuscript also showed that such flexible fragment helped the ligand to bend and to fit better its phenyl ring into the receptor cavity. 

In the case of compound **31**, two major orientations could be distinguished in the docking results (see Tables S2 and S3). The orientation 1 (pose ranked by Autodock VINA as 1 and 3 in Table S3) with the dimethylophenyl ring that formed cation-pi interactions with Lys91 or stacking with Tyr39. The second ligand orientation (poses: 2 and 5) was rotated 180 degrees and contained the dimethylophenyl ring that formed stacking interactions with Tyr118/Phe90 residues. There is a noticeable correlation between the predicted influence of compound **31** substituents in stacking interactions [49] and experimental results of Harikrishnan et al. suggesting that these interactions play an important role in its antagonism for VPAC1. Nevertheless, assessing the substituents influence on stacking interactions only in terms of electron-donating/withdrawing has proved to be insufficient in some cases [49]. For this reason, described above hypotheses on compound **31** and **41** binding modes were evaluated by molecular dynamics simulations (see Section 2.2.3). 

In comparison with ECD, the TMD allosteric binding site containing only one aromatic residue—Tyr7.57 (see Figure S10), and therefore, is rather less probable as the compound **31** and **41** binding site. Nevertheless, the molecular docking of compound **41** was also performed for this potential binding site using Autodock VINA [69]. Two poses of compound **41** that were in the agreement with the experimental data [28] were selected for the further MD study (see Section 2.2.3). Here, only hypothetical interactions of compound **41** with the TMD domain were studied in detail because compound **31** is rather too small to block the receptor activation in a similar way like other known negative allosteric modulators (NAMs) of class B GPCRs (see Section 2.2.1).

#### 2.2.3. Binding Modes of VPAC1 Antagonists Refined by Molecular Dynamics Simulations

To confirm the compound **31** and **41** binding modes obtained from molecular docking a series of short, 20 ns molecular dynamics simulations for each ligand-receptor complex was performed (see Table S3). The protein core including the ECD binding site remained stable during all simulations while its N and C terminal fragments circa 5-residues long were fully flexible because of the removal of the TMD domain. Performing a series of MD simulations including both domains would be too computationally demanding. The heavy atom RMSD of the last simulation frame computed for the full (including flexible N and C-terminus) VPAC1 ECD backbone in did not exceed 4 Å in all cases (in four cases it was below 3 Å). 

In general, ligands were very flexible during the MD simulations and compound **31** and **41** binding modes obtained from docking were preserved in only few cases, possibly because of the rather shallow binding site of the VPAC1 ECD domain (see Figure S9). The lowest heavy atom RMSD of the last simulation frame of the 20 ns simulation was obtained for two poses: 5 (compound **31**, below 2 Å) and 16 (compound **41**, below 3.5 Å) (see Table S3). The respective ligand RMSD values computed for the whole simulation trajectory were in the range: 1.5–4.0 Å (compound **31**) and 1.75–3.25 Å (compound **41**) (see Table S3).

**Figure 4:**
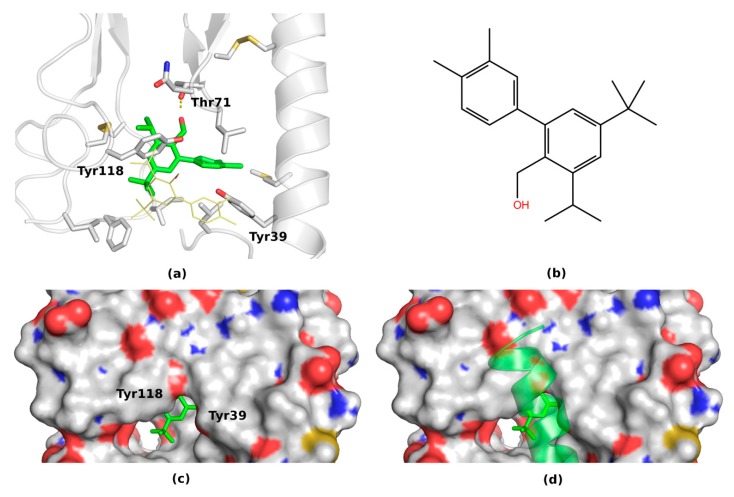
The MD-refined pose 1 (**a**) of compound **31** (**b**) containing the two-tyrosines gate (**c**) blocking the VIP binding (**d**).

Despite the high degree of the movability both ligands kept their close contact with the ECD domain forming stable binding modes at the end of simulations. Both final binding modes for **31** and **41** (see below) represented sufficient blockades for the endogenous peptide binding. The binding of the antagonist **31**, more potent than **41**, resulted in moving two polar residues: Tyr118, Tyr39 (and in some cases also the third residue - Glu36—see Table S2) to the surface of the ECD binding site (see Figure 4). Most probably, VIP binds to ECD with its mostly non-polar side (see Figure 2 and Material and Methods—Section 4.2.3) and that requires the hydrophobic residues in the binding cavity and a moderately shallow binding site fitting a helical peptide (see Figure 2 and Figure S9). Therefore, polar residues and a bulky ligand in the binding site would block VIP from binding to VPAC1 (see Figure 4c,d). 

**Figure 5:**
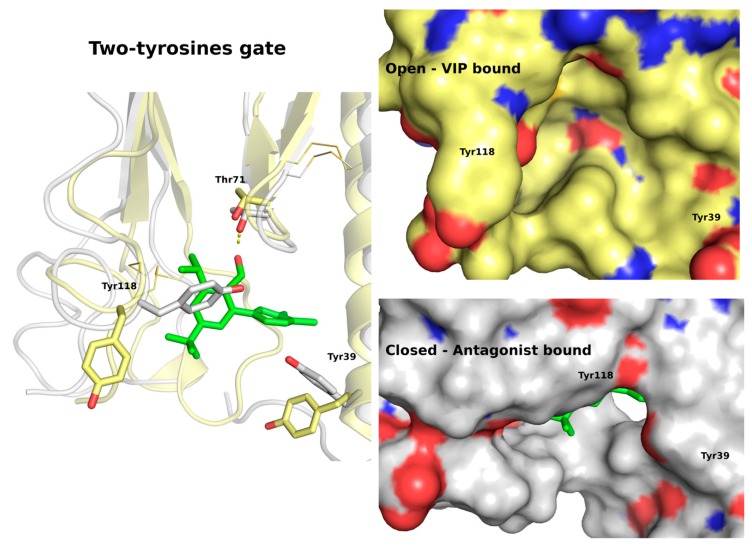
A two-tyrosines gate as the molecular basis of the VPAC1 antagonism. The peptide-bound, open conformation of VPAC1 (yellow) closes upon the antagonist binding (grey). In the closed conformation of ECD, two tyrosines: Tyr118 and Tyr39, form a gate or a steric hindrance that prevents the VIP binding.

The most stable in 20 ns simulation **31** ligand pose was the pose 5 (see Figure S11). Nevertheless, in this pose the hydroxy group of the ligand did not form any polar contacts with the receptor, except its main chain atoms. In contrast, in another relatively stable ligand pose (pose 1) the hydroxy group formed a stable hydrogen bond with Thr71 (see Figure 4). In both of these poses (1 and 5) two tyrosine residues Tyr39 and Tyr118 were closed to each other forming a gate closing upon the antagonist binding and preventing the VIP binding (see Figure 5). It was difficult to discard one of these poses based only on the 20 ns MD simulations. 

For this reason, the MD simulations of these two VPAC1 complexes (pose 1 and 5) were extended to 50 ns. As expected, the lack of stable polar interactions of the hydroxy group of compound 31 (pose 5) resulted in further changes of the ligand position (see Table S4). The ligand was moving out of the pocket, although still close to the binding site. In the case of pose 1, the ligand was kept stable during the whole 50 ns simulation, forming hydrogen bonds either with Thr71 side chain or its main chain (see Table S4 and Figure S12). Regarding the two-tyrosines gate, both residues were in close contact during the whole simulation forming a blockade for the peptide binding. However, a significant conformational variability of the N-terminal helical fragment of ECD caused by lack of the TMD domain influenced these binding site residues (see Figure S12). To further elucidate a behavior of compound **31** (and also **41**) inside the binding pocket after 50 ns, the TMD domain should be included in the system to stabilize the ECD domain.

Based on these extended MD simulations, the pose ranked as 1^st^ by Autodock VINA was selected as the most plausible for compound **31** (see Figure 4). Additionally, the two-tyrosines gate mechanism of the antagonist action was confirmed (see Figure 5), though it may involve some switching between two phenyl rings of the ligand and of Tyr39. Certainly, MD simulations including two domains of VPAC1 would provide more insights into the mechanism of action of antagonist 31, nevertheless the current study showed the most important element of it (see Figure 5). Namely, the two-tyrosines gate that prevents binding of peptide ligands by a significant change of the ECD orthosteric site regarding its polarity and shape was proposed (see Figure 5).

**Figure 6:**
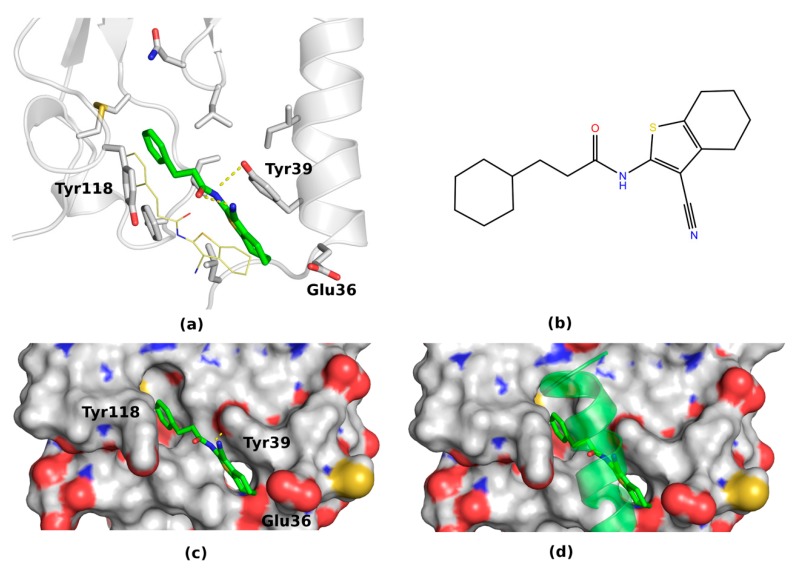
The MD-refined pose 1 (**a**) of compound **41** (**b**) containing two tyrosine residues Tyr39 and Tyr39 (**c**) in conformations that prevent the peptide binding (**d**).

The most stable in MD pose of compound **41** was presented in Figure 6. Interestingly, the only difference comparing the docking results regarding this pose is a parallel movement of the ligand towards Tyr39. After this movement, the cyanothiophene ring of compound **41** formed a stable hydrogen bond with Tyr39 on one side and was closed to Tyr118 from the other side. Thus, again the two-tyrosine gate was formed but in this case with an oxygen atom of the ligand in its middle (see Figure 6). As in the case of compound **31**, this newly formed polar gate could prevent the VIP binding (see Figure 6c,d). Other poses of compound **41** were rather unstable during MD simulations (see Table S3) and therefore less plausible.

The above described results for compound **31** and **41** still lack an adequate experimental confirmation. However, the available mutagenesis data for VPAC1 described in [14] is in agreement with these results (see Figure S4). Namely, residues that are important for the peptide binding are located in a beta turn and in a following helical turn that also includes Tyr118. This helical turn is stabilized by a disulphide bond (mutagenesis data for Cys) and three Pro residues with Gly (mutagenesis data for Gly) in between. The mutagenesis data referring to this helical turn confirms its importance, and thus, also confirms the hypothesis about the role of Tyr118 in the VPAC1 antagonism.

In addition to the above results for the ECD domain of VPAC1, two MD simulations of two complexes of VPAC1 with compound **41** inside the TMD allosteric binding site were carried out. Although it is rather unlikely that this compound binds to the allosteric site of VPAC1, the results of these MD simulations were presented in the Supplementary (Table S5). In both 30 ns simulations, the ligand positions were kept stable inside the pocket formed by TMH6 and TMH7 (see Table S5) with the heavy atom RMSD in the range of 1.0–3.5 Å – pose 1 and 1.5–6 Å (the last frame: 3.5 Å) – pose 5. The only aromatic residue Tyr7.57 was located to far from the ligand to form with it any stacking or polar interactions. Nevertheless, in the case of pose 1, the conserved among class B GPCRs serine residue S6.41 formed a hydrogen bond with the ligand. Based on these results, the pose ranked by Autodock VINA as 1^st^ seems to be more plausible than 5^th^. Provided the compound **41** acts as a negative allosteric modulator, which is rather unlikely, its antagonist effect on the receptor would be to prevent the TMH6 movement during the activation by polar interactions. The transmembrane core of VPAC1 was also stable, with the heavy atom backbone RMSD fluctuating in the range of 1.2–1.6 Å (pose 1) and 1.2–1.8 Å (pose 5). This result is also a verification of the constructed VPAC1 homology model that was described in this manuscript. A further verification of this VPAC1 homology model in its inactive conformation was carried by extending the MD simulation to 80 ns (see Material and Methods).

## 3. Discussion and Conclusions

Although this computational study is preliminary, it is still unique among very few computational studies of VPAC and PACAP receptors [73], despite their clinical importance. Based on the results presented in this manuscript, a conclusion could be formed, that the two VPAC1 antagonists, known to date, **31** and **41** most probably block the receptor orthosteric site located in its ECD domain rather than modulate the receptor activation via the TMD allosteric site. Nevertheless, compound **41** was also stable in the allosteric site of VPAC1 during the 30 ns (and 80 ns) MD simulation. 

The molecular description of the mechanism of the VPAC1 antagonism by small-molecules was described in detail. Most probably it involves the two-tyrosines gate closing upon binding of the antagonist. The mechanism of action of two antagonists **31** and **41** was explained based on the molecular modeling in accordance with the experimental data from the functional assays and mutagenesis data. Based on the presented results from the molecular dynamics and molecular docking studies, new modifications of **31** and **41** antagonists can be introduced to increase their potency for VPAC1. Thus far, no improvements in the potency of these antagonists have been obtained despite a number of tested derivatives in the study of Harikrishnan et al. The usage of VPAC1 as a drug target in the treatment of common inflammatory diseases together with problems associated with gastrointestinal side effects and still a small number of bioavailable, non-peptidic antagonists emphasize the importance of this class B G protein-coupled receptor.

Among limited studies regarding the PACAP receptors family, one recent study by Takasaki et al. [74] described small-molecule antagonists of PAC1R that were identified by virtual screening. It was shown experimentally that they behave as potent and specific antagonists of PAC1R as they do not modulate the activity of VPAC1 or VPAC2. Their binding site was predicted to involve the N-terminal fragment of PAC1R ECD. More particularly, in a sub-pocket formed by Leu80, Phe81, Ile83, Gly91, Val92, Pro107, Ala112, Cys113, Ser120, and Glu121, which was described by us as highly diverse region among known-to-date class B structures (see Figure 1). The same region was selected by us for the molecular docking and during MD simulations we observed significant conformational changes of it leading to the closure of the two-tyrosines gate upon antagonist binding (see Figure 5). Results of Takasaki et al. and ours are thus consistent with the two-domain binding model of ligand/receptor interaction in which the specificity of the ligand is ensured by precise interactions with the ECD. Moreover, recently, Sarkar et al. [75] used the concept of two domains for the VPAC1 receptor and have suggested new small-molecules to be its antagonists that bind to TMD. However, in absence of any experimental data, it is difficult to conclude if these molecules are indeed specific and/or active and to predict if these compounds will behave as agonists or antagonists. 

## 4. Materials and Methods

### 4.1. Homology Modeling and Molecular Docking

#### 4.1.1. An Inactive Transmembrane Conformation of VPAC1 for Testing Allosteric Modulators

An inactive conformation of the TMD domain of VPAC1 was constructed using the allosteric antagonist-bound crystal structures of GLP-1R and GCGR (PDB id: 5XEZ, 4L6R, 5EE7, 5VEW) [76,77] (see Figure S13 for the sequence alignment). To date, GLP-1R is the most similar to VPAC1 class B GPCR of a known crystal structure that could be used as a template. A sequence identity computed with ClustalOmega [65] between VPAC1 (Uniprot id: P32241) and GLP-1R (P43220), GCGR (P47871), CRFR1 (P34998), CALCR (P30988) sequences is: 40.00, 37.44, 30.45, 26.05, respectively. The VPAC1 model was built using the well-known procedure GPCRM, developed by author, described in a number of publications [76,77,78,79,80,81] and cited in a few benchmarking studies [82,83,84,85]. 

Based on the GLP-1R crystal structure containing an antagonist molecule (5VEW) a corresponding potential allosteric binding site in VPAC1 was defined (see Figure S10) and confirmed by SiteMap computations [67,68] (see Table S1). The experimentally confirmed small-molecule non-peptidic antagonist **41** was prepared with MGLtools [86] and docked using Autodock VINA [69]. Two poses were selected for molecular dynamics simulations. 

#### 4.1.2. An ECD Domain of VPAC1 for Testing Antagonists

To reconstruct an extracellular domain of VPAC1 the VPAC2 ECD crystal structure (PDB id: 2X57) and full (ECD and TMD) crystal structures of GLP-1R (5NX2, 5VAI) and GCGR (5YQZ) receptors were used (see below – Section 4.1.3). To investigate interactions of antagonists only with the ECD domain, we removed the whole TMD domain from the VPAC1 model (see Figure S3). 

The ligand docking of experimentally confirmed antagonists **31** and **41** to the ECD binding site that was defined by the VPAC1-peptide interactions (see Figure 2) was performed. Additionally, the binding site selection was confirmed with SiteMap (see Table S1) and comparison with other solved-to-date experimental structures of class B GPCRs (see Figure 1). Ligands were prepared with MGLtools. Four receptor structures: the VPAC2 crystal structure (PDB id: 2X57) and three models of the VPAC1 ECD domain built using: 5NX2, 5VAI and 5YQZ template structures were used for the docking study (see Figures S3 and S9). In all four cases the binding site was the same—based on 5VAI and 5YQZ crystal structures that contained peptide analogs in the orthosteric binding site of GLP-1R and GCGR receptors, respectively.

For docking, we used the flexible-receptor flexible-ligand docking procedure in Autodock VINA. The box size was set to 20 Å and it covered only the mentioned above orthosteric binding site in ECD (see Figure 2). For molecular dynamics studies we selected poses of the highest affinity for the receptor, the best fitting the binding site cavity (see Table S3). A total number of four poses for compound **31** and four poses for compound **41** was selected for further MD studies.

#### 4.1.3. Active and Partly Active Two-Domains Models of VPAC1 with VIP

A full model of VPAC1 was built using: the VPAC2 crystal structure of ECD (2X57) and 5NX2, 5VAI and 5YQZ template structures that included both, ECD and TMD domains of GLP-1R and GCGR receptors (see Figure S1). In the case of two latter templates structures instead included peptide agonists we used the VIP model obtained using its NMR structure (2RRI), the only one that has been deposited in PDB thus far. All model building was performed using GPCRM for TMD domains and MODELLER for a full VPAC1 model including ECD (5NX2, 5VAI, 5YQZ) and its complex with peptides (5VAI, 5YQZ) and G proteins (5VAI). The corresponding multiple sequence alignments were presented in Figures S14–S16. The models were validated in the additional 100 ns MD study (see Section 4.2.3, Figures S17 and S18).

### 4.2. Molecular Dynamics Simulations of VPAC1 and Its Complexes With Antagonists

#### 4.2.1. MD Simulations of the ECD Domain Complexes

Complexes of ECD domains with both antagonists were prepared for molecular dynamics simulations using the CHARMM-GUI web server [87] including the information about three disulphide bonds. The ligands parameterization was carried out using the CHARMM-GUI web server, based on their mol2 files generated with LigPrep, Schrodinger LLC, 2018 [68]. The periodic rectangular water box (TIP3P) was fitted to the protein size with the edge distance 10 Å. Each system was neutralized by addition of Na^+^ and Cl^−^ ions, with ionic concentration of 0.15 M. The number of atoms in each simulation was equal to circa 42,900 atoms. The Charmm36 force field was used in each simulation. The equilibration step included 10,000 steps (500 steps – 1ps) of the steepest descent minimization followed by 25,000 steps of the conjugated gradients minimization. The equilibration simulation was performed in NVT using the Langevin dynamics (303.15 K). The time integration step in the equilibration and production runs was set to 2 fs. The production run in NPT was performed using the Langevin piston Nose-Hoover method (1 bar, 303.15 K) and lasted 20 ns (extended in two cases to 50 ns) for each system. The GPU version of NAMD was used for all MD simulations. Conformational fluctuations of ECD stabilized after about 0.5–1 ns of production runs with backbone RMSD equal to about 2–2.5 Å. The further conformational changes of ECD (see Figure S12) were either mainly due to its C and N terminals that were lacking interactions with TMD (2–3.5 Å) or due to moving away the central, bottom fragment of the binding cavity with two Phe residues with simultaneous breaking of a short helical turn inside (2–5.5 Å). Other fragments of the ECD domain, forming beta sheets and an alpha helix that were stabilized by disulphide bonds and proline residues did not change its conformation to a significant extent.

#### 4.2.2. MD Simulations of the TMD Domain Complexes

Complexes of TMD domains with both antagonists were prepared for molecular dynamics simulations using the CHARMM-GUI web server including the information about the conserved EC2 disulphide bond. Each of two simulation systems, containing the ligand-receptor complex embedded within the membrane (OPM-oriented [88]) and solvated (TIP3P) was neutralized by addition of Na+ and Cl- ions, with ionic concentration of 0.15 M. The membrane was formed by POPC and cholesterol molecules with proportion of 5:1. The number of atoms in each simulation was equal to circa 92000 atoms. The Charmm36 force field was used in each simulation. The equilibration step included six stages, lasting for: 20 ps (steepest descent minimization) + 50 ps (conjugated gradients minimization), 50 ps, 50 ps, 200 ps, 200 ps, and 200 ps. During six equilibration stages atomic position restraints were gradually released, e.g., for the protein backbone atoms: from 10 (1^st^ stage) to 0.1 kcal·mol^−1^·Å^−2^ (6^th^ stage). Only for the first stage of equilibration, a 1 fs time integration step was used. The first two stages were performed in NVT, the next four in NPT (1 bar, 303.15 K) using the Langevin dynamics. The production run in NPT was performed using the Langevin piston Nose-Hoover method (1 bar, 303.15 K) and lasted 30 ns for each system (one simulation was extended to 80 ns to validate the inactive VPAC1 model—see Figure S19). The GPU version of NAMD was used for all MD simulations. Conformational fluctuations of the transmembrane core of the receptor stabilized after about 5 ns of production runs with the heavy atom backbone RMSD equal to about 1.6 Å and did not change after extending the simulation to 80 ns. 

#### 4.2.3. MD Simulations of an Active Conformation of VPAC1.

VPAC1 models obtained as described in Section 4.1.3 were subjected to 100 ns MD simulation runs for validation. Models were prepared for molecular dynamics simulations using the CHARMM- GUI web server including the information about the conserved EC2 disulphide bond. All systems were embedded within the POPC membrane (OPM-oriented) and solvated (TIP3P) was neutralized by addition of Na^+^ and Cl^−^ ions, with ionic concentration of 0.15 M. The number of atoms in simulations of VPAC1 models based on 5NX2, 5VAI and 5YQZ were equal to circa 140,000, 273,000, 120,000 atoms, respectively. The Charmm36 force field was used in each simulation, since this force field was tested in various aspects of full-atom simulations of biological systems composed of membrane proteins and lipids, as well as small-molecule compounds. Charmm36 was also applied in a number of studies regarding GPCRs [89]. The equilibration step included six stages, lasting for: 20 ps (a steepest descent minimization), 50 ps (a conjugated gradients minimization), 50 ps, 50 ps, 200 ps, 200 ps, and 200 ps. During the six equilibration stages, atomic position restraints were gradually released, e.g., for the protein backbone atoms: from 10 (the first stage) to 0.1 kcal·mol^−1^·Å^−2^ (the sixth stage). Only for the first stage of equilibration, a 1 fs time integration step was used. The first two stages were performed in NVT, the next four in NPT (1 bar, 310K) using the Langevin dynamics. The production run in NPT was performed using the Langevin piston Nose-Hoover method (1bar, 310K) and lasted 100 ns for each system applying 2 fs timestep. The GPU version of NAMD was used for all MD simulations. Conformational fluctuations of the transmembrane core of the receptor stabilized after about 25–50 ns of the production runs with the heavy atom backbone RMSD equal to about 2.6, 1.6, and 2.3 Å for VPAC1 models based on 5VAI, 5YQZ and 5NX2 templates, respectively (see Figure S18). Additionally, we observed that positions of the selected peptide residues that were depicted in Figure 2b were stable during the MD simulations with RMSD equal to 4 Å (see Figure S19).

## Figures and Tables

**Table 1:** Lack of selectivity in binding of endogenous peptides to its class B GPCRs subtypes.

Peptide Hormone ^1^	Primary GPCR Target	Other GPCR Targets
Secretin	SCTR	VPAC1, VPAC2 [39]
Amylin [34]	AMY_1_, AMY_2_, AMY_3_	CTR
Adrenomedullin	AM_1_, AM_2_	CGRP receptor
Adrenomedullin 2	AM_2_	AM_1_
GHRH (GRF)	GHRHR	VPAC1, VPAC2 ^2^
PACAP ^3^	PAC1, VPAC1, VPAC2	melanocortin receptors (class A) [40]
VIP	VPAC1, VPAC2	PAC1
CRF [41,42] ^4^	CRFR1 ^5^	CRFR2
Urocortin1 [41]	CRFR1, CRFR2	
Urocortin2 [41]	CRFR2	CRFR1
Urocortin3 [41]	CRFR2	CRFR1

^1^ Here, we presented only endogenous peptides observed in humans; ^2^ The higher affinity towards VPAC receptors has been observed for GRF analogs [43,44,45]; ^3^ There are two PACAP peptides differing in length in humans: PACAP-27 and PACAP-38; ^4^ CRF1 and CRFR2 receptors can also be activated by non-human urotensin I (CRF2 > CRF1) and sauvagine (CRF2 > CRF1) [42]; ^5^ Another receptor for CRF (CRFR3) has also been found, but only in catfish thus far [46].

## Supplementary Materials

Supplementary materials can be found at https://www.mdpi.com/1422-0067/20/18/4348/s1.

## Abbreviations

VIP	Vasoactive intestinal peptide
VPAC1	Vasoactive intestinal peptide receptor 1
VPAC2	Vasoactive intestinal peptide receptor 2
PACAP	Pituitary adenylate cyclase-activating peptide
PAC1R	Pituitary adenylate cyclase-activating peptide type I receptor
GCGR	Glucagon receptor
GLP-1R	Glucagon-like peptide-1 receptor
CGRP	Calcitonin gene-related peptide receptor
RAMP	Receptor-activity modifying protein
GPCR	G protein-coupled receptor
ECD	Ectodomain (extracellular domain) of class B GPCRs
TMD	Transmembrane domain of GPCRs
EC2	Extracellular loop 2 of GPCR
NMR	Nucleic Magnetic Resonance
MD	Molecular Dynamics
RMSD	Root-mean-square deviation of atomic positions
NVT	An ensemble of a constant number of particles, volume and temperature
NPT	An ensemble of a constant number of particles, pressure and temperature
