# Supplementary material for: A Molecular Dynamics Study of Vasoactive Intestinal Peptide Receptor 1 and the Basis of Its Therapeutic Antagonism
Source: Int J Mol Sci. 2019 Sep 5;20(18):4348. doi: 10.3390/ijms20184348 (PMC6770453; doi:10.3390/ijms20184348)
Supplement: Supplementary file 1 [file ijms-20-04348-s001.pdf]

International Journal of
Molecular Sciences

A Molecular Dynamics Study of Vasoactive Intestinal Peptide Receptor 1 and the Basis of its Therapeutic Antagonism

Dorota Latek [1,*], Ingrid Langer [2], Krystiana Krzysko [3], Lukasz Charzynski [3]

1 Faculty of Chemistry, University of Warsaw, 02-093 Warsaw, Poland; dlatek@chem.uw.edu.pl
2 Institut de Recherche Interdisciplinaire en Biologie Humaine et Moléculaire (IRIBHM), Université libre de Bruxelles, B-1070 Brussels, Belgium
3 Faculty of Physics, University of Warsaw, 02-093 Warsaw, Poland
* Correspondence: dlatek@chem.uw.edu.pl

Supplementary materials

This file includes figure S1–S19 and table S1–S5 that corresponds to the main manuscript text.

Tables

Table S1. Results of the SiteMap computations for both, ECD and TMD domains of VPAC1.

	SiteScore	Dscore	Size (number of site points)	Enclosure	Hydrophilic	Hydrophobic
ECD domain						
**Site 1[1]**	**0.764**	**0.742**	**52**	**0.628**	**0.959**	**0.511**
Site 2	0.677	0.644	39	0.591	0.913	0.179
TMD domain						
**Site 1 (allosteric)**	**1.049**	**1.087**	**92**	**0.751**	**0.814**	**1.354**
Site 2 (orthosteric)	1.024	1.044	188	0.729	1.024	0.502
Site 3	0.809	0.839	25	0.714	0.183	3.704
Site 4	0.722	0.724	28	0.637	0.478	2.190
Site 5	0.671	0.622	31	0.647	0.887	0.819

[1]Here, two binding sites of VPAC1, one in ECD and the other in TMD were bolded. Both of them were selected for the purpose of the study and both of them were assigned the highest SiteScore and Dscore by SiteMap.

Table S2. The Autodock VINA-approximated binding affinity for compounds **31** and **41** towards VPAC1 and VPAC2 ECD domains.

	VPAC2 – 2X57	VPAC1 based on 5NX2 (partially active)	VPAC1 based on 5VAI (fully active)	VPAC1 based on 5YQZ (fully active)
Compound **31**				
Orientation 1	None found, the ligand on the surface (the lowest score: -8.3)	-7.4[1]	-9.6	-8.6
Orientation 2	-7.2	-7.3	-9.3	-8.6
Compound **41**				
Orientation 1	-6.8	-6.6	-9.0	-7.4
Orientation 2	-7.8	-7.0	-7.7	-8.1

[1] Here, the Autodock VINA score for the first-ranked pose of a given orientation is provided.

Table S3. Results of MD simulations of VPAC1-antagonists complexes. Here, the ligand orientation obtained in molecular docking was shown in yellow lines.

Compound / pose no / major orientation	Autodock VINA score	MD-refined hypothetical binding mode	Ligand RMSD	Receptor ECD RMSD
**Compound 31 – pose 1, orientation 1**	**-9.6**	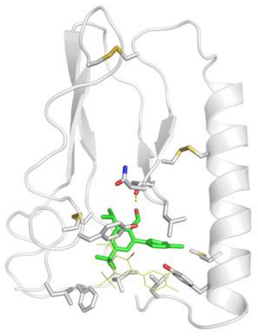	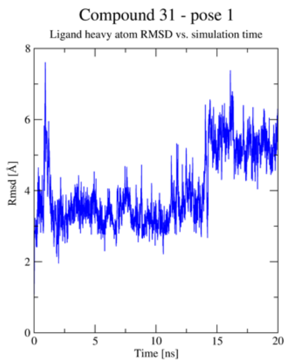	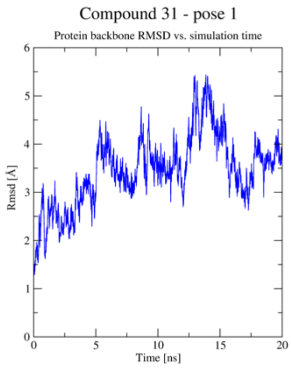
Compound 31 – pose 2, orientation 2	-9.3	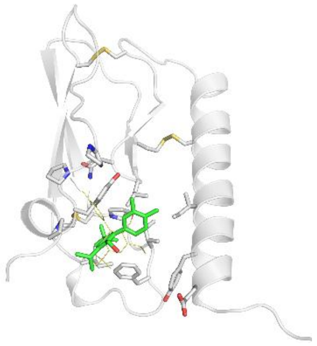	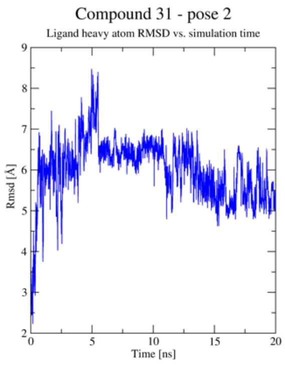	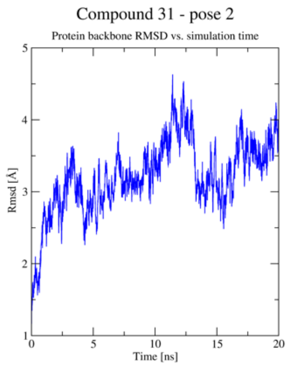

Compound 31 – pose 3, orientation 1	-9.3	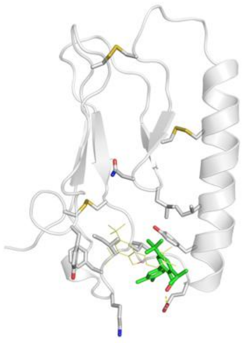	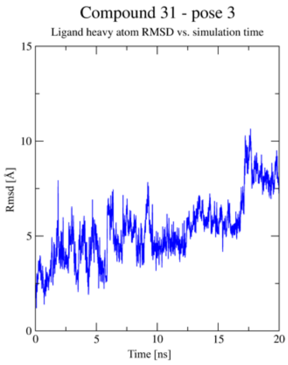	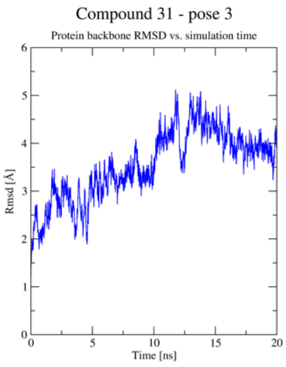
**Compound 31 – pose 5, orientation 2**	**-8.8**	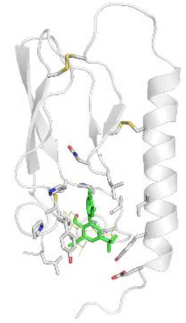	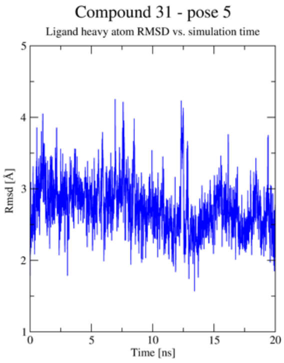	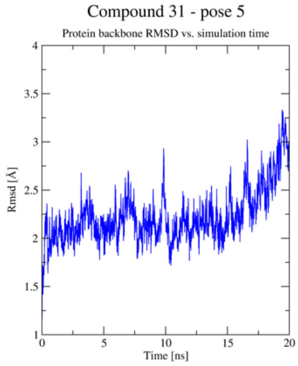
Compound 41 – pose 1, orientation 1	-9.0	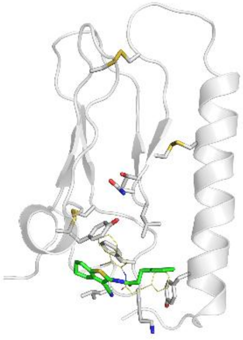	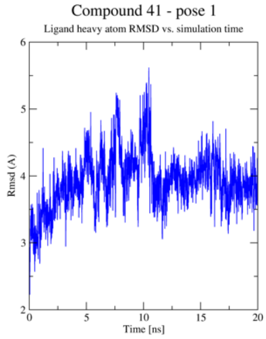	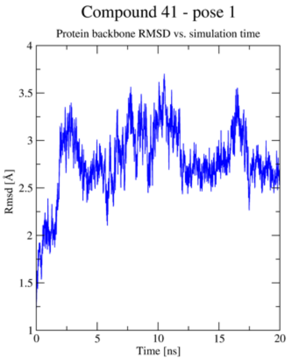
Compound 41 – pose 6 (5YQZ), orientation 2	-7.8	Ligand pose outside the binding site. 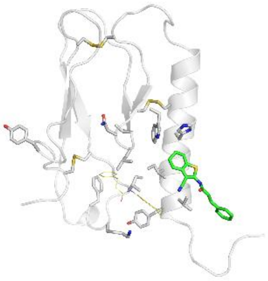	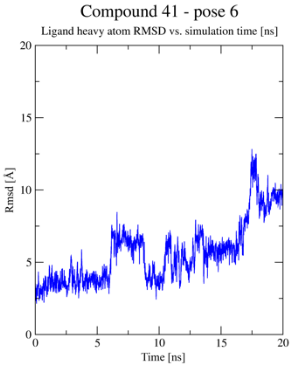	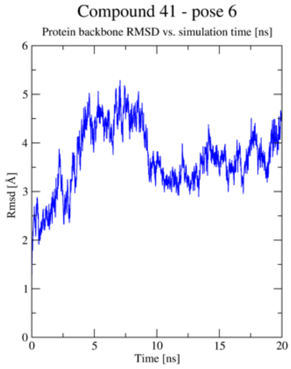

Compound 41 – pose 8, orientation 2	-7.7	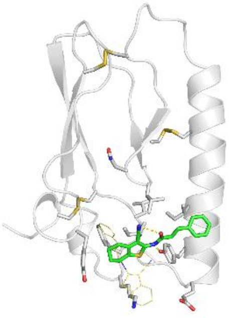	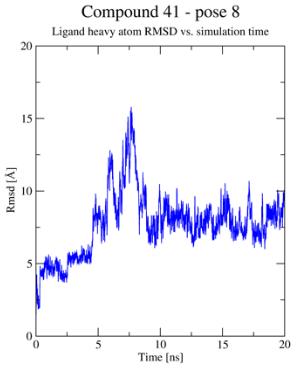	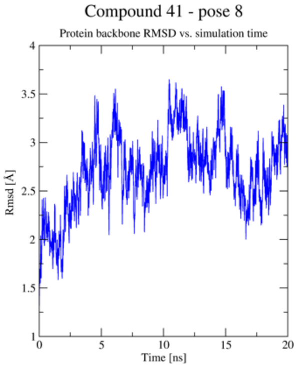
**Compound 41 – pose 16, orientation 2**	**-7.2**	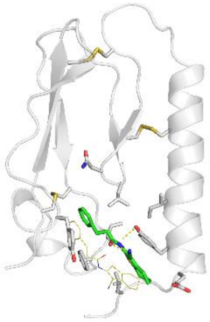	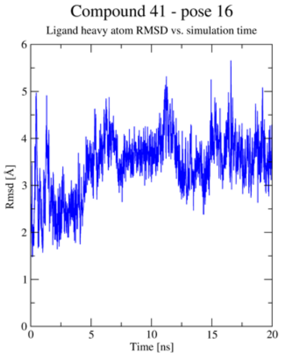	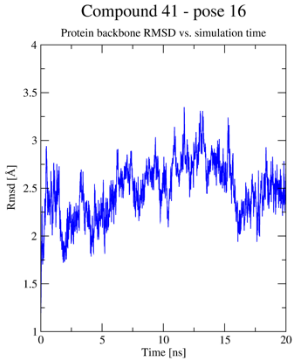

Table S4. Results of the extended to 50 ns (shown last 30 ns) MD simulations of compound **31** complexes (poses 1 and 5). Here, the last snapshot of the previous 20 ns MD simulation was shown in yellow.

Pose no.	MD-refined binding mode	Ligand RMSD	Receptor ECD RMSD
Pose 1	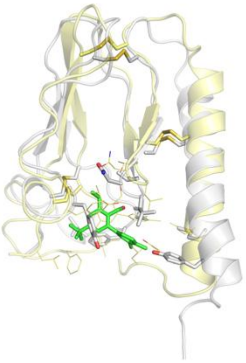	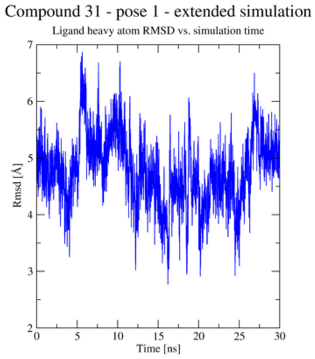	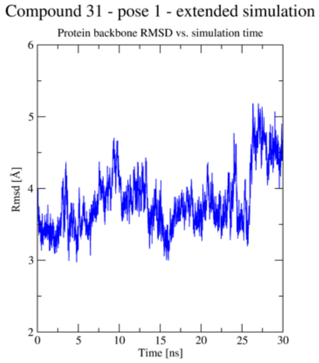
Pose 5	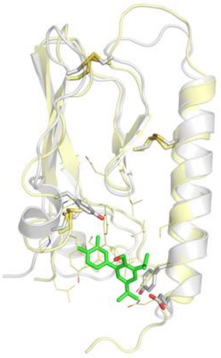	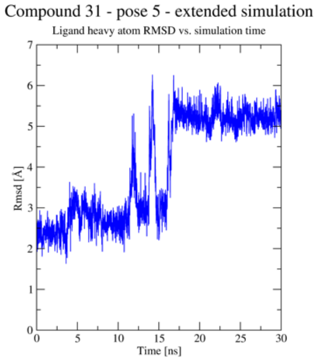	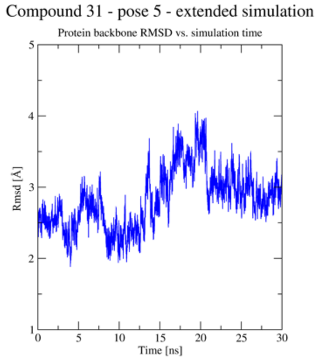

Table S5. Results of MD simulations of compound **41** complexes – the TMD region of VPAC1. Here, the ligand orientation obtained in molecular docking was shown in yellow lines.

Pose no.	Autodock VINA score	MD-refined binding mode	Ligand RMSD	Receptor TMD RMSD
Pose 1	-9.1	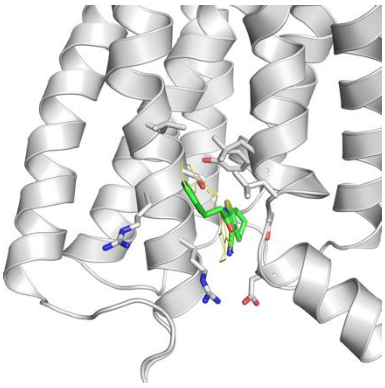	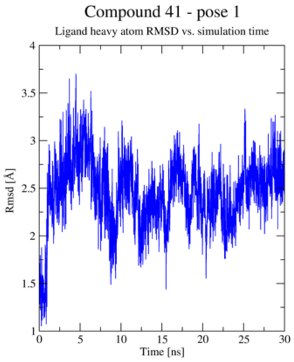	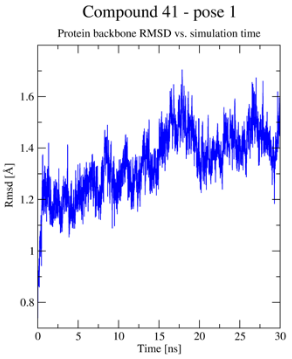
Pose 5	-7.8	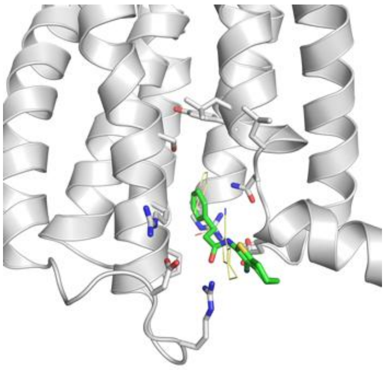	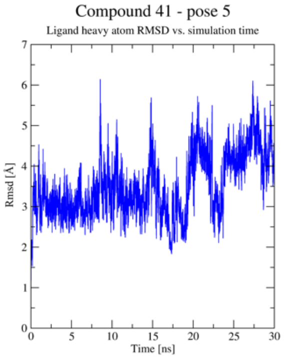	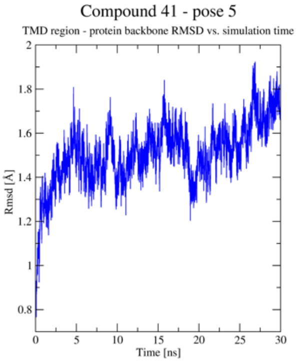

Figures

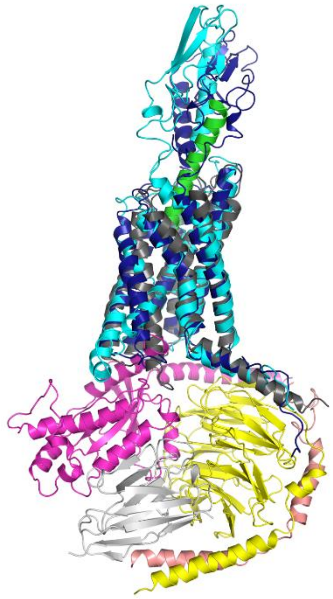

Figure S1. The activation mechanism of class B GPCRs – superposition of active, partly active and inactive conformations of VPAC1. Here, we used VPAC1 models based on 5VAI (blue), 5NX2 (dark blue) and a set of inactive structural templates (grey). The active VPAC1 model based on 5VAI (GLP-1R) was constructed together with VIP (green) and G protein subunits (grey, magenta, yellow, pink). TMH6 is subject to the greatest conformational change – a sharp kink in the middle that enables interactions with the G alpha subunit (magenta). The ectodomain of VPAC1 deflects without any major conformational changes of secondary structure enabling interactions with a peptide hormone.

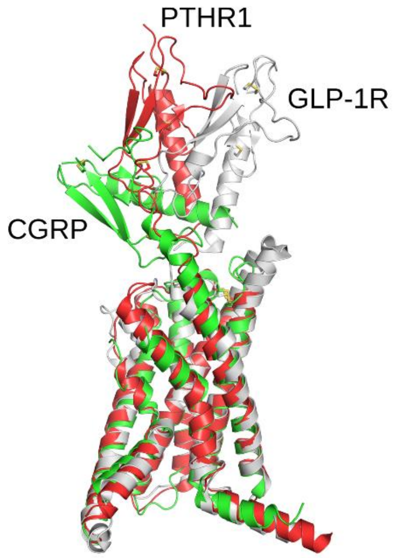

Figure S2. The comparison of conformational states of the activated class B receptors. The ECD domain of CGRP (CLR+RAMP) is the closest to its TMD domain because of additional interactions with the extracellular part of RAMP (not shown). Structures of GLP-1R (grey, PDB id: 5VAI), PTHR1 (red, PDB id: 6NBF), CGRP (green, PDB id: 6E3Y) were superimposed in their TMD regions to observe differences in the relative positions of ECD and TMD domains. TMD domains of shown here receptors undergo similar conformational changes upon the activation, e.g., the TMH6 deformation.

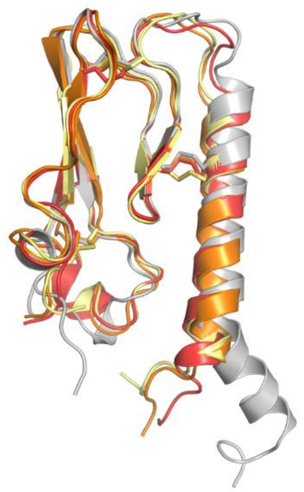

Figure S3. Comparison of the VPAC2 structure of ECD (grey, PDB id: 2X57) and models of the VPAC1 ECD domain constructed using: 5NX2 (yellow), 5VAI (orange) and 5YQZ (red) template structures.

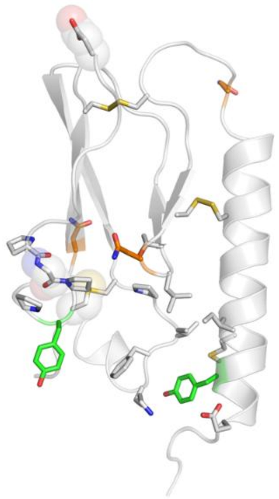

Figure S4. The site-directed mutagenesis data for VPAC1 shown in its model of the extracellular domain. Orange residues are glycosylated (Asn58, Asn69, Asn100), residues shown in the spheres representation are crucial for the peptide binding (Asp107, Gly116, Cys122) which was confirmed by the site-directed mutagenesis. Other binding site residues are shown with the sticks representation (green – the two-tyrosines gate – see the main manuscript text). Here, we showed only three first residues out of five (Asp107, Gly116, Cys122 and Lys127 and Gln135) that were confirmed experimentally as important for the peptide binding. The remaining two residues are in the flexible region joining two domains that were not shown here.

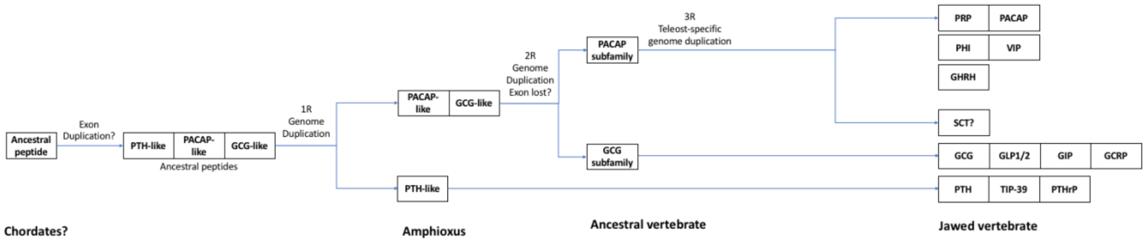

Figure S5. A proposed evolutionary model of the PACAP-related genes

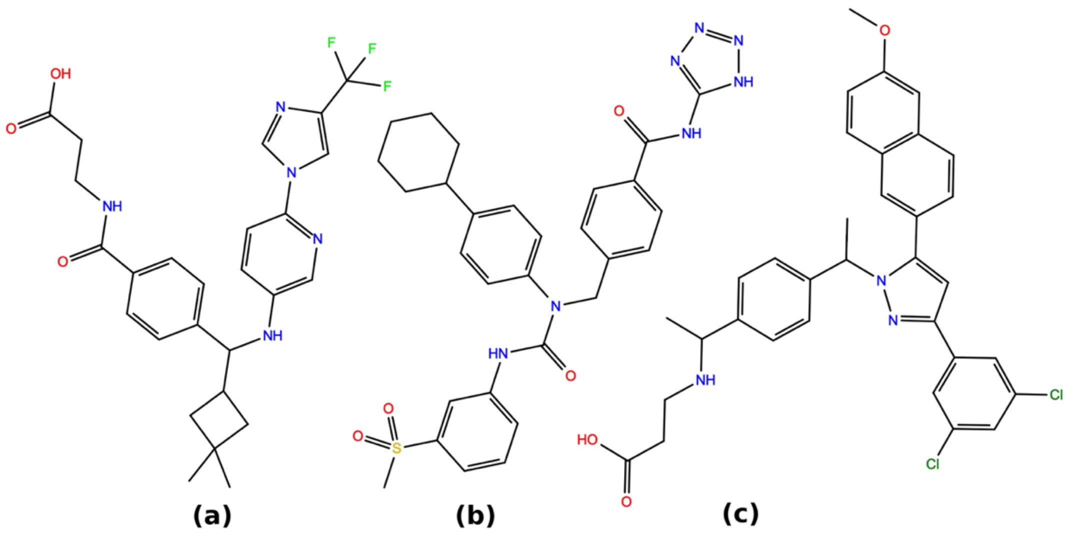

Figure S6. Negative allosteric modulators of class B GPCRs present in solved to date experimental structures. (a) PF-06372222 (GLP-1R – 5VEW), (b) NNC0640 (GCGR – 5XEZ), (c) MK-0893 (GCGR – 5EE7). All of them possess a distinct V-shape that blocks TMH6 from bending.

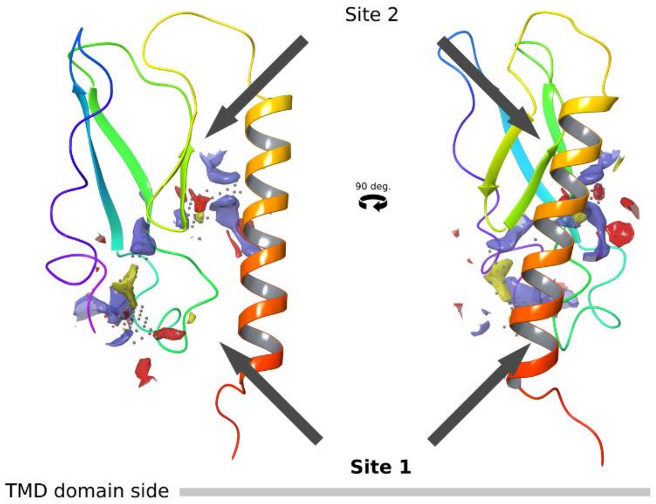

Figure S7. Potential ECD binding sites of VPAC1 detected by SiteMap. The binding site selected for the current study as more probable was bolded. The binding site representation is as provided by SiteMap (Schrodinger, LLC): grey spheres – site points, surfaces – interactions sites (yellow – hydrophobic, blue – hydrogen bonding donor, red – hydrogen bonding acceptor sites). The color scheme – red-to-blue (N-termini as red).

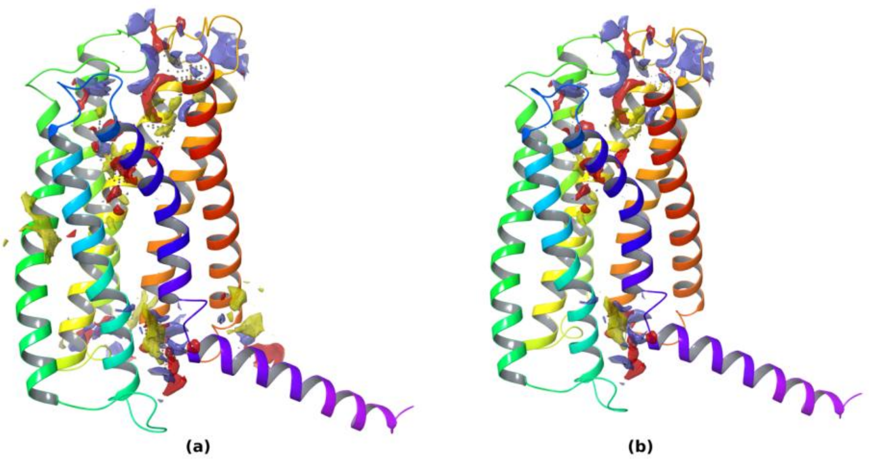

Figure S8. All potential TMD binding sites of VPAC1 detected by SiteMap (a). Two major binding sites (b) that were confirmed by solved-to-date structures of class B GPCRs: the orthosteric site for the peptide binding and the allosteric site for the allosteric modulators binding. The binding site representation was the same as in Figure S7.

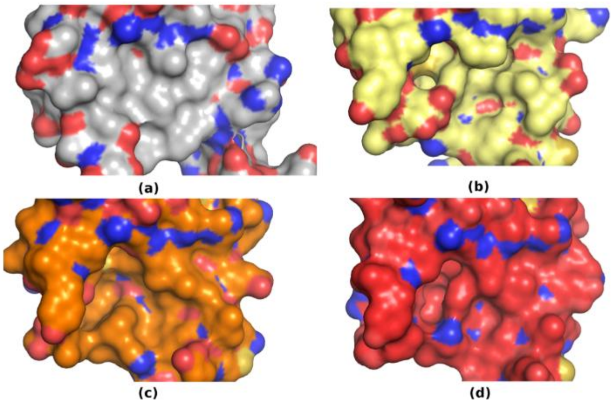

Figure S9. Comparison of the binding site cavity located in ectodomains of VPAC2 (**a**) and VPAC1 (**b**, **c**, **d**). (b) – The VPAC1 model based on 5NX2, (c) – the VPAC1 model based on 5VAI and (**d**) – the VPAC1 model based on 5YQZ. The VPAC2 binding cavity is the shallowest and hydrophobic. The most spacious cavity-like binding site was observed for the VPAC1 model based on 5VAI.

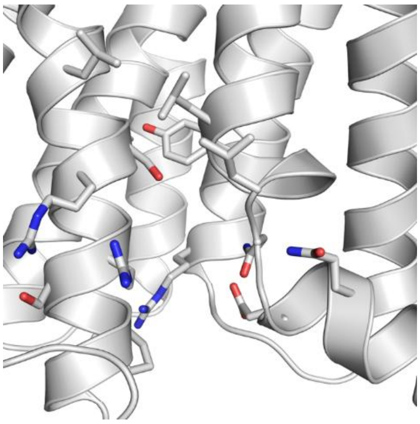

Figure S10. The allosteric binding site of VPAC1 located in its transmembrane domain. Here, we presented the VPAC1 model in its inactive conformation, constructed using a set of inactive template structures. The binding site is mostly occupied by polar residues with a single aromatic residue Tyr7.57 in the middle.

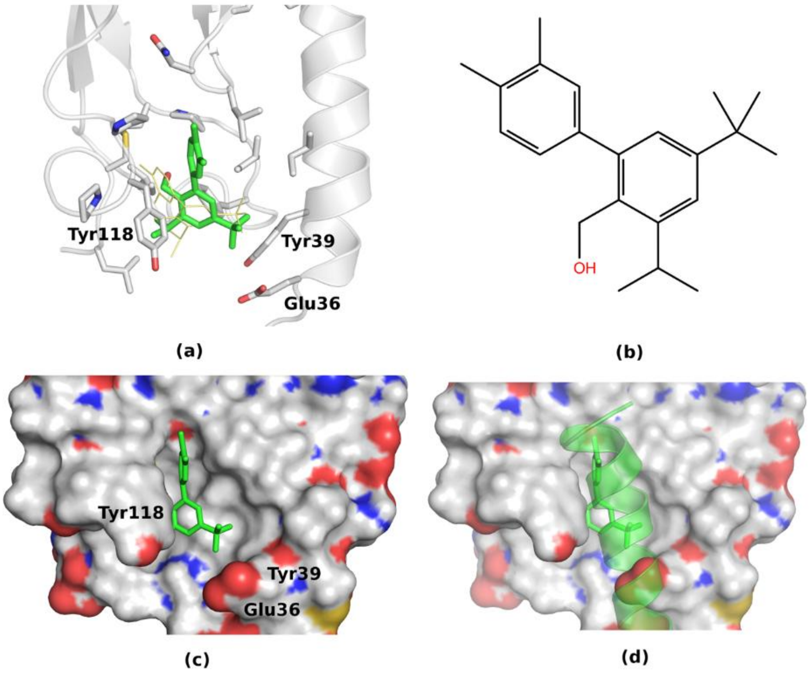

Figure S11. The MD-refined pose 5 of compound **31** (the 20 ns simulation).

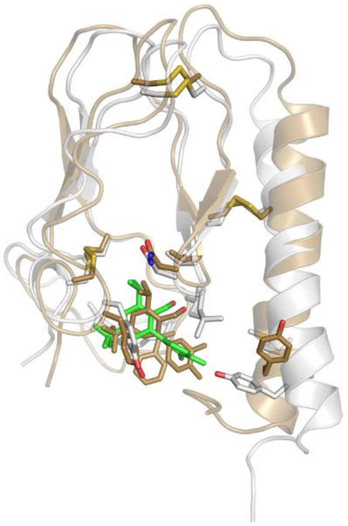

Figure S12. Results of the extended (from 20 ns to 50 ns) simulation of the compound **31** and VPAC1 complex (pose 1). Here, the last frame of the simulation (50 ns) is shown in brown and the last frame with extended N-terminal helical fragment of ECD (~45 ns) is shown in grey. A further extension of this MD simulation should be carried out in the presence of the TMD domain because of too significant mobility of N and C-terminal fragment that might influence the binding site and crucial tyrosine residues (Tyr118 and Tyr39) positions.

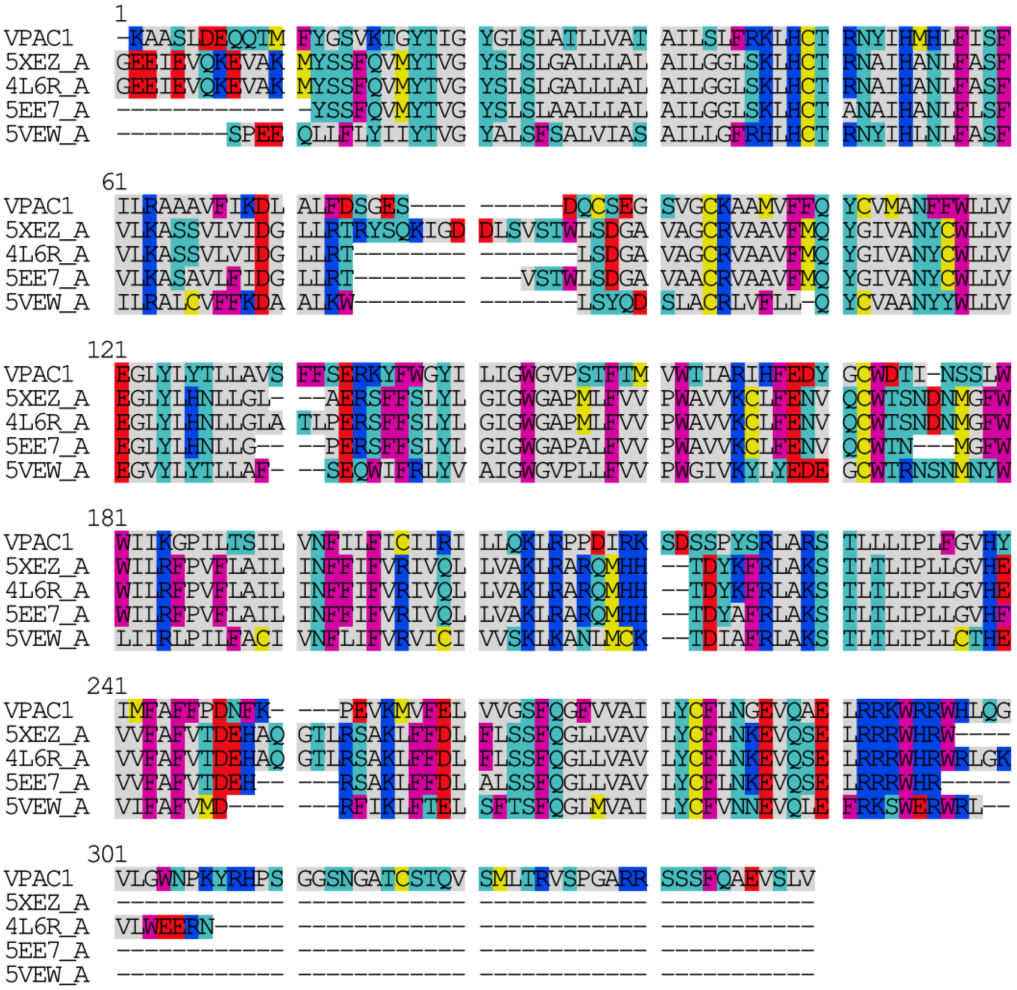

Figure S13. A multiple sequence alignment that was used to build an inactive VPAC1 model.

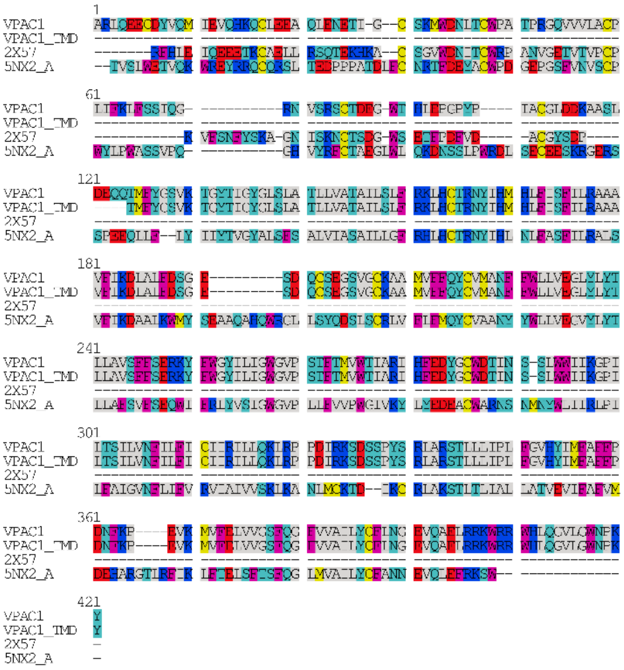

Figure S14. A multiple sequence alignment that was used to build an active VPAC1 model based on the GLP-1R structure (5NX2) including the ECD domain.

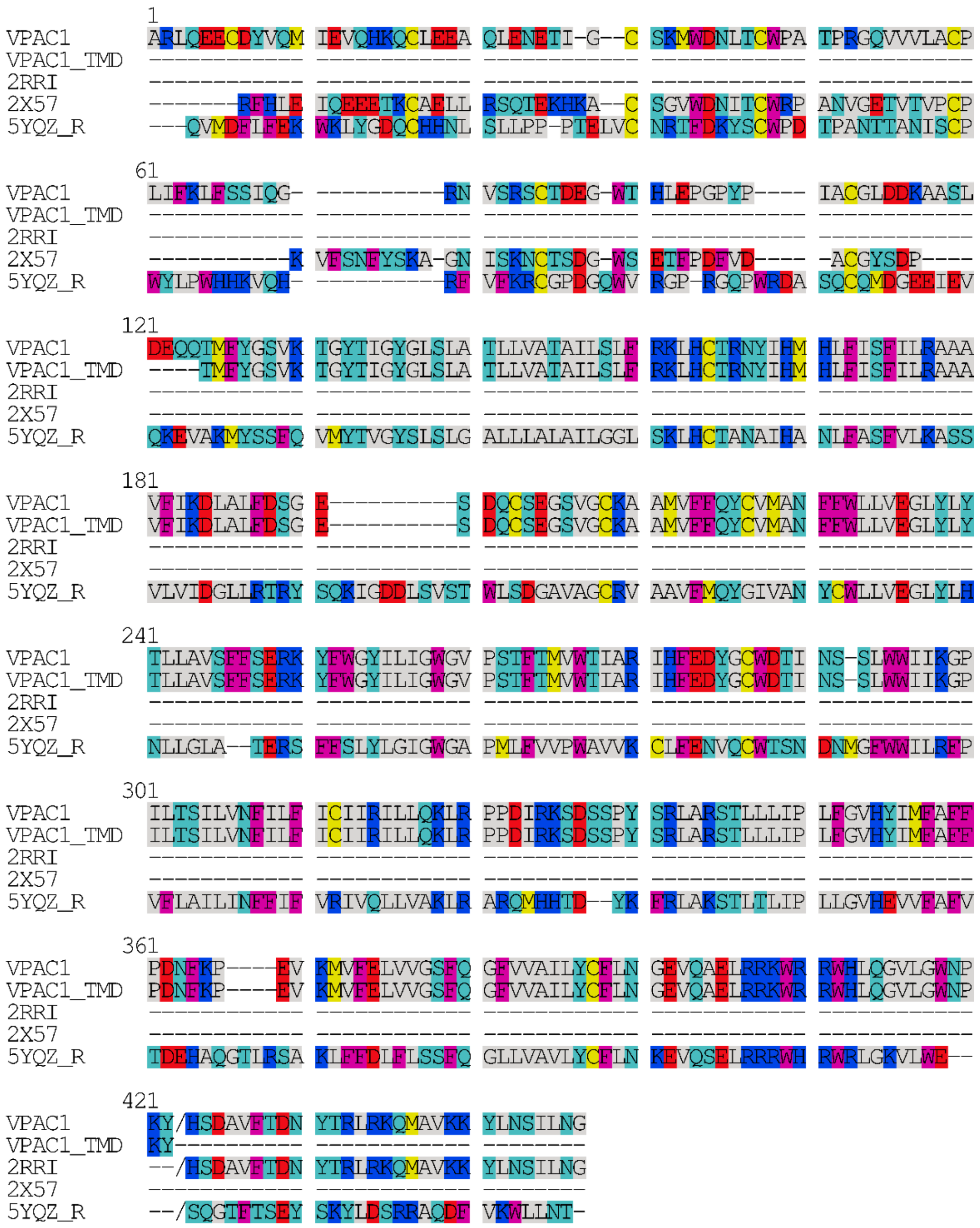

Figure S15. A multiple sequence alignment that was used to build an active VPAC1 conformation model based on the GCGR structure (5YQZ) including the ECD domain and VIP.

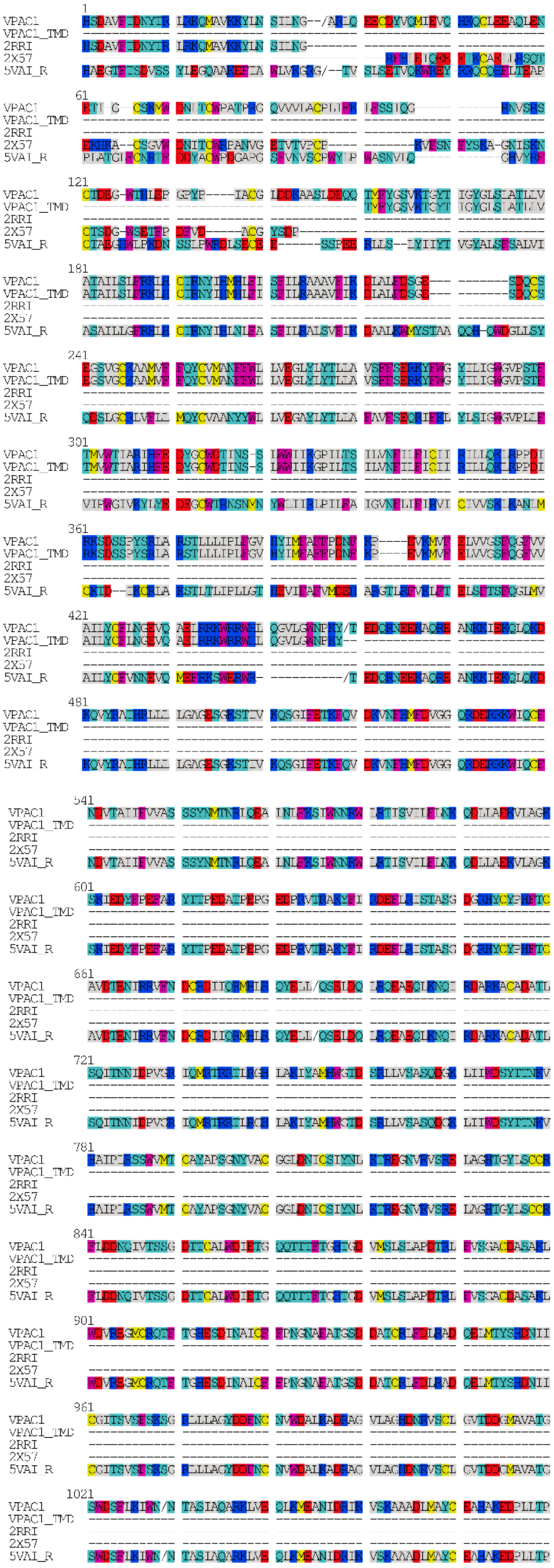
VPAC1
VPAC1_TMD
2RRI
2X57
5VAI_R
1
61
121
181
241
301
361
421
481
541
601
661
721
781
841
901
961
1021

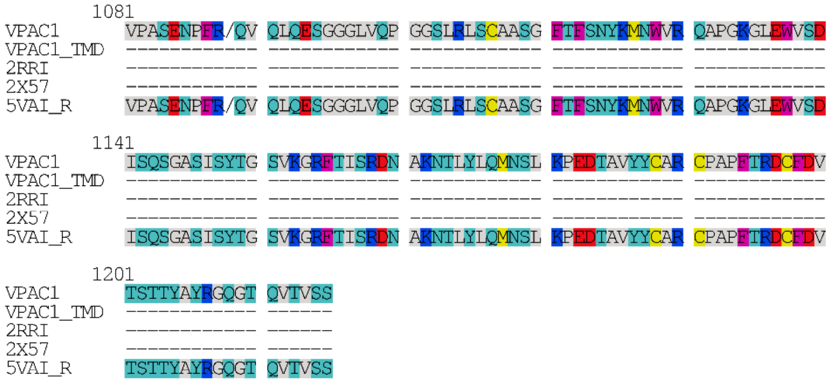

Figure S16. A multiple sequence alignment that was used to build an active VPAC1 conformation model based on the GLP-1R structure (5VAI) including the ECD domain, VIP and G protein subunits.

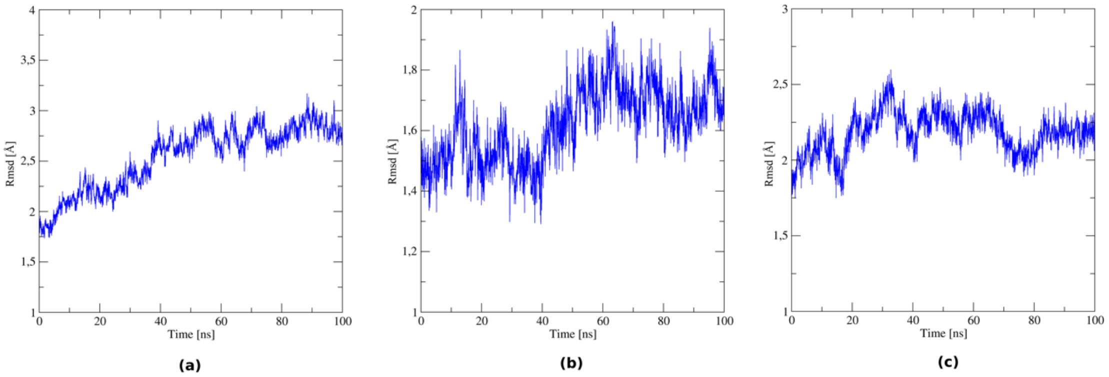

Figure S17. A validation of VPAC1 models presented in this study by all-atom 100 ns MD simulations. Here, protein backbone RMSD of the transmembrane helices was presented, similarly to data presented in Table A5. (a) The VPAC1 model based on 5VAI. Here, VIP and G protein subunits were included in the simulation system. (b) The VPAC1 models based on 5YQZ. Here, the VIP was included in the simulation system. (c) a VPAC1 model based on 5NX2. Here, only the receptor was included in the simulation system. The least change in the receptor structure was observed for (b), most probably due to a well-defined secondary structure of the N-terminal helical fragment of TMD joining with ECD. The largest changes in the receptor structure were observed for (a), due to the presence of the whole G protein subunits complex in the simulation system.

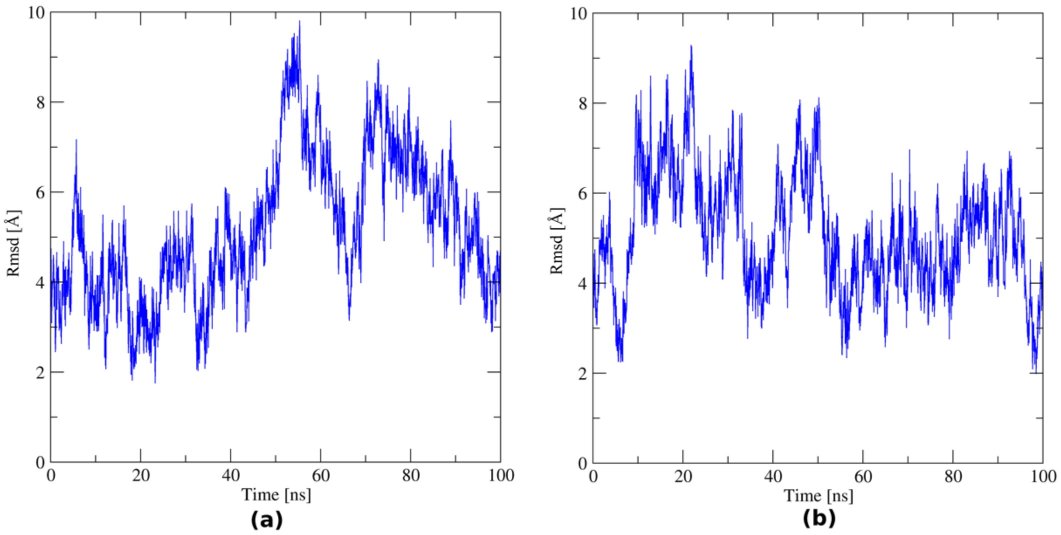

Figure S18. Results of 100 ns MD simulations of the VIP – VPAC1 complex. Here, two models of the VIP-VPAC1 complex were presented: (a) the model based on the 5VAI template structure, (b) the model based on the 5YQZ. Heavy atom RMSD values were computed for the interface residues of VIP that interact with ECD (see Figure 2b in the main manuscript text, the upper part, residues shown in sticks). In both cases, this peptide interface remained stable.

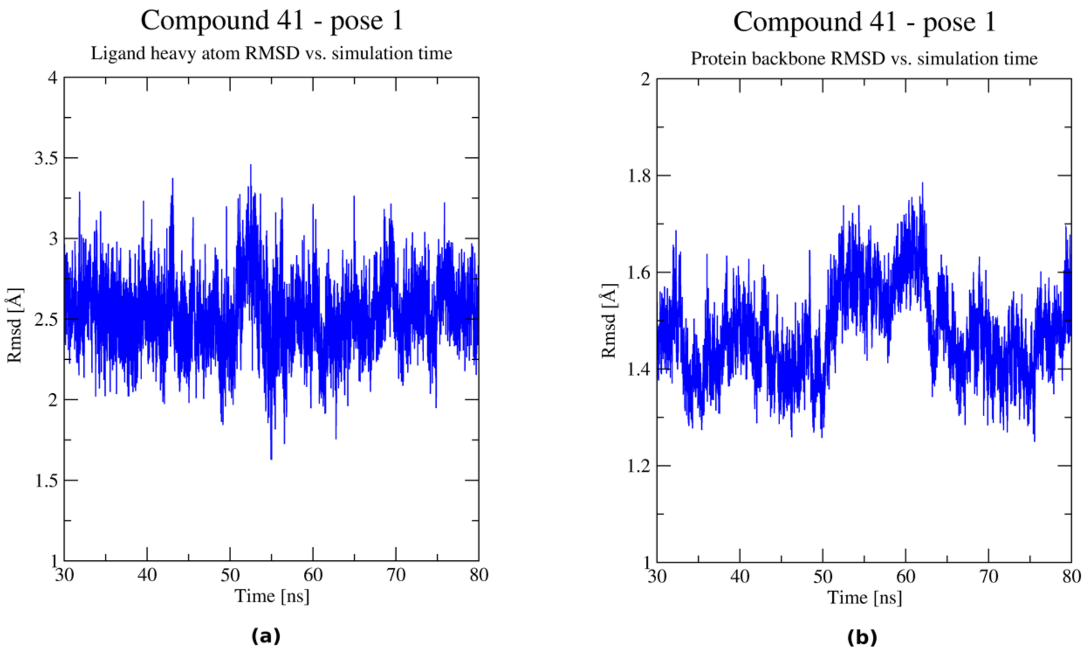

Figure S19. Results of the extended simulation (to 80 ns) of the compound **41** in complex with TMD of VPAC1 (pose 1 - see Table S5). Here, ligand and protein RMSD was computed, similarly to data presented in Table S5.
